# Nuclear VPS35 attenuates NHEJ repair by sequestering Ku protein

**DOI:** 10.1186/s10020-025-01288-1

**Published:** 2025-06-09

**Authors:** Luping Zhang, Yonghong Nie, Tuo Tang, Yanji Lu, Wenlong Li, Xian Hong, Qiang Li, Aixue Zheng, Yongpei Li, Jianwen Zhou, Li Fan, Tao Wang, Zhihui Deng

**Affiliations:** 1 Laboratory of Protein Structure and Function, Institute of Medicine and Pharmacy, Qiqihar Medical University, Qiqihar, Heilongjiang 161006 China; 2 Department of Medical Technology, Qiqihar Medical University, Qiqihar, Heilongjiang 161006 China; 3 Laboratory of Molecular Biology, Institute of Medicine and Pharmacy, Qiqihar Medical University, Qiqihar, Heilongjiang 161006 China; 4 Heilongjiang Provincial key Laboratory of Precise Diagnosis and Neuropsychological Regulation of Mental Disorders, Qiqihar, Heilongjiang 161006 China

**Keywords:** VPS35, Non-homologous end joining, DNA repair, Ku

## Abstract

Vacuolar protein sorting-associated protein 35 (VPS35), a pivotal constituent of the retromer complex, mediates the retrograde trafficking of endosomal cargoes in the cytoplasm. Intriguingly, VPS35 displays a dual localization pattern, residing both in the cytoplasm and the nucleus, but its nuclear role remains elusive. In this study, we unravel a nuclear function of VPS35, demonstrating it impedes DNA repair by inhibiting non-homologous end joining (NHEJ). Mechanistically, VPS35 interacts with the Ku protein, sequestering it away from DNA damage sites. Consequently, nuclear VPS35 halts the activation of DNA-PKcs, hindering the recruitment of XLF and DNA-Ligase 4, ultimately suppressing NHEJ efficiency. Furthermore, in response to DNA damage, VPS35 dissociates from Ku protein and orchestrates a strategic relocation from the nucleus to the cytoplasm. Thus, our findings suggest VPS35 attenuates NHEJ repair by restricting Ku protein availability at DNA damage sites, offering a potential avenue for fine-tuning DNA repair efficiency.

**Supplementary Information:**

The online version contains supplementary material available at 10.1186/s10020-025-01288-1.

## Introduction

Pathological DNA double strand breaks (DSBs) are regarded as the most perilous DNA lesions due to their potential to jeopardizes genomic integrity. In the context of DSB repair, non-homologous end joining (NHEJ) and homologous recombination (HR) represent two fundamental pathways that cells rely on to restore genomic stability. HR is a precise but complex process. It requires a homologous template, usually the sister chromatid during the S and G2 phases of the cell cycle, to guide the repair of the damaged DNA. HR ensures high fidelity repair by utilizing the intact sequence information from the template. However, its reliance on a homologous template limits its effectiveness during certain stages of the cell cycle, such as G1, when sister chromatids are not yet available. On the other hand, NHEJ is a more rapid and efficient process. It involves the direct ligation of broken DNA ends, often without the need for extensive sequence homology. As a template-independent DNA repair mechanism, NHEJ works throughout the cell cycle and is responsible for the majority of DSB repair (Tan et al. 2023).

Initiation of NHEJ involves the binding of the Ku70/Ku80 heterodimer, also known as Ku protein, to the newly formed DSB ends. Subsequently, the Ku70/Ku80 heterodimer recruits the DNA-dependent protein kinase catalytic subunit (DNA-PKcs) to constitute the DNA-PK complex. This complex serves as a central component in NHEJ by marshaling other downstream NHEJ factors to DSB ends. Additionally, DNA-PKcs, acting as a kinase, triggers the phosphorylation of the chromatin factors H2AX and KRAB associated protein 1 (KAP1) following DSB induction, leading to chromatin decondensation (Lu et al. 2019; Nakamura et al. 2010; Ziv et al. 2006). This decondensation of chromatin in the vicinity of the DSB creates a conducive environment for the recruitment of DNA damage response (DDR) proteins such as XRCC4-like factor (XLF) and DNA-Ligase 4, ultimately enhancing the NHEJ efficiency (Price and D’Andrea 2013).

In recent years, several Ku associated proteins have been added to the NHEJ repertoire besides the well-known NHEJ factors. These proteins include Paralog of XRCC4 and XLF (PAXX) (Liu et al. 2017; Ochi et al. 2015), Modulator of retroviral infection (MRI) (Arnoult et al. 2017; Hung et al. 2018), ERCC excision repair 6 like 2 (ERCC6L2) (Francica et al. 2020; Liu et al. 2020), ZNF384 (Singh et al. 2021), Forkhead box protein L2 (FOXL2) (Jin et al. 2020), cyclic GMP-AMP Synthase (cGAS) (Zhang et al. 2024a), Wiskott-Aldrich Syndrome Protein and SCAR Homolog (WASH) (Wang et al. 2022) and FAM21 (Wang et al. 2024). Some of these proteins serve as downstream factors of Ku protein, recruited by Ku to orchestrate DNA repair events. For instance, ERCC6L2 localizes to DNA damage sites through its interaction with Ku protein, facilitating the recruitment of MRI, XRCC4, and DNA-Ligase 4 for directional end-joining (Liu et al. 2020). cGAS, the well-known cytosolic DNA sensor, interacts with Ku80 to stabilize DNA-PKcs by enhancing the interaction between DNA-PKcs and the deubiquitinase USP7, thereby promoting canonical NHEJ (Zhang et al. 2024a). Our research has shown that WASH and its partner FAM21 interact with Ku70/Ku80 to enhance DNA DSB repair by expediting the activation of DNA-PKcs following DNA damage (Wang et al. 2022, 2024). Additionally, other Ku associated proteins act as regulators, modulating the accumulation of Ku protein at DNA DSBs. For example, PAXX and ZNF384 augment the association of Ku protein with damaged chromosomes, thus facilitating NHEJ repair (Liu et al. 2017; Ochi et al. 2015; Singh et al. 2021). In contrast, FOXL2 interacts with Ku protein to inhibit the formation of Ku heterodimer, thereby inhibiting NHEJ repair (Jin et al. 2020). Moreover, MRI plays dual roles in the regulation of DNA repair. In the G1 phase, it interacts with Ku protein to promote the localization of Ku-containing DDR proteins at DSB-associated chromatin (Arnoult et al. 2017; Hung et al. 2018); whereas in the S and G2 phases, MRI obstructs the initiation of the NHEJ process, potentially by competing for Ku binding with other NHEJ factors (Arnoult et al. 2017).

Vacuolar protein sorting-associated protein 35 (VPS35) is a key component of the retromer complex that is responsible for retrograde transport of endosomal cargoes. Previous studies have demonstrated that cytoplasmic VPS35 interacts with the unstructured c-terminal tail of FAM21 to recruit the WASH regulatory complex (SHRC) to endosomal subdomains enriched with retromer (Guo et al. 2024; Harbour et al. 2012; Jia et al. 2012; Romano-Moreno et al. 2024). Despite VPS35 shows dual localization in both the cytoplasm and nucleus, its precise nuclear function remains enigmatic (Deng et al. 2015). Our previous study has shown that nuclear FAM21 promotes the WASH recruitment to DNA damage site through its interaction with Ku protein (Wang et al. 2024). This finding prompts us to hypothesize that VPS35, the partner of FAM21, may also play a role in the intricate process of DNA repair.

In this study, we demonstrate that VPS35 engages in a functional interaction with Ku protein, effectively sequestering the Ku protein away from DNA damage sites. Consequently, VPS35 halts the activation of DNA-PKcs, impeding the timely recruitment of XLF and DNA-Ligase 4, ultimately diminishing the efficiency of NHEJ. Notably, upon the induction of DNA damage, VPS35 dissociates from Ku protein and orchestrates a strategic translocation to the cytoplasm, freeing Ku protein to carry out its indispensable role in DNA repair. Our findings reveal a previously unidentified functional aspect of VPS35 in the context of DNA repair and underscore the dynamic and intricate interplay between VPS35 and Ku protein.

## Materials and methods

### Cells, reagents and plasmids

HEK-293T and HeLa cells were purchased from National Infrastructure of Cell Line Resource. The generation of VPS35^WT^ + Vector (VPS35^WT^), VPS35^KO^ + Vector (VPS35^KO^) and VPS35^KO^ + VPS35^Rescue^ (VPS35^Res^) HeLa cells have been described (Hong et al. 2022). All cells were cultured in DMEM medium supplemented with 10% fetal bovine serum. Etoposide (E1383), BrdU (B5002), protein A/G agarose beads (P5906 and P7700) and antibody against Flag (F1804) were purchased from Sigma. Bleomycin sulfate (HY-17565) was purchased from MedChemExpress. Anti-flag affinity magnetic beads (BMFLAG-1) and streptavidin magnetic beads (BMSA500) were purchased from BioMag. Antibody against VPS35 (ab226180) and p-DNA-PKcs (ab18192) were purchased from Abcam. Antibody against DNA-PKcs (12311S) was purchased from Cell Signaling Technology. Antibody against γH2AX (05-636) was purchased from Millipore. Antibodies against Ku70 (10723-1-AP), Ku80 (16389-1-AP and 66546-1-Ig) and GAPDH (60004-1-Ig) were purchased from Proteintech Group. Antibody against RPA1 (T200948) was purchased from Zenbio. Antibody against Histone H3 (711055), Goat anti-Mouse IgG (H+L) Cross-Adsorbed Secondary Antibody, Alexa Fluor 488 (A-11001), Goat anti-Rabbit IgG (H + L) Highly Cross-Adsorbed Secondary Antibody, Alexa Fluor 488 (A-11034) and Goat anti-Rabbit IgG (H+L) Cross-Adsorbed Secondary Antibody, Alexa Fluor 568 (A-11036) were purchased from ThermoFisher. Mouse Anti-Rabbit IgG-HRP (sc-2357) and m-IgGκ BP-HRP (sc-516102) were from Santa Cruz Biotechnology. Mouse IgG (A7028) and Rabbit IgG (A7016) were purchased from Beyotime. Lipofectamine 3000 (L3000015) was purchased from Invitrogen. Plasmids and EJ5-GFP (44026), DR-GFP (26475) and pCBASecI (26477) were purchased from Addgene. The plasmids pSpCas9(BB)-2A-Puro (PX459) V2.0, PCI2-HA-mCherry, PCI2.Flag, pLenti6.3.Flag.MCS, pCMS3.H1p/HA-YFP, pCMS3.H1p.shVPS35/HA-YFP and pCMS3.H1p.shVPS35/HA-YFP-VPS35 were a generous gift from Professor Daniel D. Billadeau (Mayo Clinic College of Medicine, MN, USA).

### Plasmid construction

The sgRNA/Cas9 expression construct with an RNA guide sequence (GTA GGA CAA AAA CAA GCT TA) targeting human VPS35 gene and plasmid pLenti6.3.Flag.VPS35 have been described (Hong et al. 2022). The plasmids YFP-XLF, YFP-DNA-Ligase 4 (YFP-LIG4), mCherry-Ku70, YFP-NLS.WASH and YFP-FAM21.Δ219N have been described (Wang et al. 2022). The plasmid pG68 was constructed by inserting a 223 bp duplex DNA fragment, which includes a series of BbvCI restriction sites, into the pUC18 vector using EcoRI and HindIII, as described in a previous study (Vidal-Eychenié et al. 2013). To obtain the plasmid PCI2.Flag.VPS35, VPS35 coding sequence was amplified by PCR using the pCMS3.H1p.shVPS35b/HA.YFP.VPS35 plasmid as the template and cloned into the PCI2.Flag vector. To obtain the plasmid pCMS3.H1p.shVPS35b/HA.YFP-NLS.linker.VPS35 (hereafter referred to as YFP-NLS.VPS35), a nuclear localization signal (NLS) tagged VPS35 coding sequence was amplified by PCR using the pCMS3.H1p.shVPS35b/HA.YFP.VPS35 plasmid as the template and cloned into the pCMS3.H1p.shVPS35b/HA.YFP vector. To obtain the pLenti6.3.Flag.NLS.linker.VPS35 plasmid, the NLS.linker.VPS35 sequence was amplified by PCR using pCMS3.H1p.shVPS35b/HA.YFP-NLS.linker.VPS35 as the template and inserted into the pLenti6.3.Flag.MCS vector. All primers are listed in Table S1.

### Generation of stable cell lines

Generation of stable cell linesVPS35^KO^ HEK-293T cells were generated using CRISPR/Cas9 technique. The sgRNA/Cas9 expression construct was transiently transfected into HEK-293T cells. Twenty-four hours after transfection, cells were treated with 2 µg/mL puromycin for 48 h prior to being plated into 96-well plates to acquire individual clones. VPS35^KO^ HEK-293T cells were validated by immunoblot. NLS.VPS35 overexpression (NLS.VPS35^OE^) cells, VPS35 overexpression (VPS35^OE^) cells and their negative control counterpart (Vector^OE^) cells were generated using a lentiviral method. To produce lentiviral particles, HEK-293T cells were co-transfected with pLenti6.3.Flag.NLS.linker.VPS35, pLenti6.3.Flag.VPS35 or pLenti6.3.Flag.MCS empty vector with psPAX2 and pMD2.G. Forty-eight hours after transfection, the lentiviral particles were obtained by harvesting the media from the cells. HEK-293T cells were transduced with the indicated lentiviral particles, and were selected using 10 µg/mL blasticidin for 10 days. NLS.VPS35^OE^, VPS35^OE^ and Vector^OE^ cells were validated by immunoblot.

### Cell viability assay

Cells were seeded into a 96-well plate at 4 ×10^3^ cells/well and cultured for 24 h. Then cells were treated with the indicated concentrations of etoposide or bleomycin for 48 h. Following this treatment, CCK-8 solution (MCE, 244902) was added to each well and incubated for 3 h. The absorbance was then measured at 450 nm using a Tecan Spark microplate reader.

### Immunofluorescence

Cultured cells grown in glass-bottom dishes were fixed with 4% paraformaldehyde, permeabilized with 0.15% Triton X-100, and blocked with immunofluorescence blocking buffer (5% goat serum, 1% glycerol, 0.1% BSA, 0.1% fish skin gel and 0.04% sodium azide in PBS buffer). Primary antibodies including γH2AX (05-636, Millipore; 1:500 dilution), VPS35 (ab226180, Abcam; 1:500 dilution), p-DNA-PKcs S2056 (ab18192, Abcam; 1:500 dilution), Ku70 (10723-1-AP, Proteintech Group; 1:500 dilution), and Ku80 (16389-1-AP, Proteintech Group; 1:500 dilution), were diluted in blocking buffer and applied overnight at 4 °C. After 5 times washing with PBS, cellular specimens received Goat anti-Mouse IgG (H+L) Cross-Adsorbed Secondary Antibody, Alexa Fluor 488 (A-11001, ThermoFisher; 1:500 dilution), Goat anti-Rabbit IgG (H+L) Highly Cross-Adsorbed Secondary Antibody, Alexa Fluor 488 (A-11034, ThermoFisher; 1:500 dilution) or Goat anti-Rabbit IgG (H+L) Cross-Adsorbed Secondary Antibody, Alexa Fluor 568 (A-11036, ThermoFisher; 1:500 dilution) through 60 min ambient incubation under light-protected conditions. Nuclear staining was achieved using Hoechst 33342. Images were captured with either a Zeiss LSM-710 or Nikon AXR confocal microscope and subsequently analyzed with ImageJ software.

### Neutral comet assay

Cells were treated with 100 µM etoposide or 200 µg/mL bleomycin for 1 h and allowed to recover for 30 min under standard growth conditions. Subsequently, cells were harvested and resuspended at a concentration of 2×10^5^ cells/mL in PBS buffer. The neutral comet assay was performed using the CometAssay^®^ Kit (4250-050-K, R&D Systems) following the manufacturer’s protocol. Briefly, molten Comet LMAgarose was cooled to 37°C and mixed with cell suspension at a 10:1 (v/v) ratio. Fifty microliters of the cell-agarose mixture were pipetted onto CometSlide and allowed to gel at 4°C for 30 min. Slides were immersed in pre-cooled (4°C) Lysis Solution for 1 h and then in 4°C 1×Neutral Electrophoresis Buffer for 30 min. Electrophoresis was conducted with 4°C 1×Neutral Electrophoresis Buffer, applying 21 volts (1 V per cm) for 35 min to allow DNA fragment migration. Following electrophoresis, slides were immersed in DNA Precipitation Solution for 30 min at room temperature, then in 70% ethanol for 30 min, and dried at 37°C for 15 min. Dried slides were stained with SYBR Green in the dark for 30 min, rinsed briefly with water, and 37°C dried. Fluorescent images were captured using either a Zeiss LSM-710 or Nikon Ni-U microscope. The calculation of tail moments was performed automatically using the OpenComet plugin of ImageJ software.

### Immunoprecipitation

For G0/G1 phase synchronization, cells were starved in serum-free DMEM for 24 h preceding bleomycin exposure. Cultured cells were exposed to either vehicle control or DNA-damaging agents (100 µM etoposide or 200 µg/mL bleomycin) for 2 h. Post-treatment cells were collected and lysed in ice-cold lysis buffer (20 mM HEPES pH 7.2, 50 mM Potassium Acetate, 200 mM D-sorbitol, 1 mM EDTA, 0.1% Triton X-100, 0.5 mM PMSF, 2 mM Na_3_VO_4_ and proteinase inhibitors). After 30 min incubation on ice, lysates were clarified by centrifugation at 12,000×g for 10 min. For co-immunoprecipitation assays, cleared supernatants were incubated with the indicated antibodies in the presence or absence of 0.2 mg/mL EtBr overnight at 4℃ with rotation. Immune complexes were captured using protein A/G agarose beads (1 h, 4℃), followed by five sequential washes with chilled lysis buffer. Bound proteins were eluted in 1×Loading buffer for subsequent immunoblot analysis. For Flag-tagged protein interactome analysis, whole-cell lysates were incubated with anti-flag affinity magnetic beads overnight at 4℃. Precipitates were then washed and subjected to mass spectrometry.

### Cytoplasmic and nuclear protein extraction

Following treatment termination, cells were harvested and washed with ice-cold Buffer A (10 mM HEPES, 1.5 mM MgCl₂, 10 mM KCl, 0.5 mM DTT). The pelleted cells were subsequently resuspended in hypotonic lysis Buffer B (10 mM HEPES, 1.5 mM MgCl_2_, 150 mM KCl, 0.1% NP-40, 0.5 mM DTT, 0.5 mM PMSF, 2 mM Na_3_VO_4_ and proteinase inhibitors) for 10 min incubation on ice. After centrifugation (6,500 rpm, 3 min, 4℃), the supernatant containing cytoplasmic components was carefully aspirated while the nuclear pellets underwent two cycles of purification washes with Buffer A supplemented with protease inhibitors. The washed nuclei were then subjected to hypertonic extraction using Buffer C (10 mM HEPES, 25% glycerol, 420 mM NaCl, 1.5 mM MgCl_2_, 0.2 mM EDTA, 0.5 mM DTT, 0.5 mM PMSF, 2 mM Na_3_VO_4_ and proteinase inhibitors) with 30 min ice incubation. Following centrifugation (12,000 rpm, 10 min, 4℃), the clarified nuclear extract was collected and diluted 1:3 (v/v) with ice-cold Buffer A containing protease inhibitors prior to subsequent immunoblot analysis.

### Proximity ligation assay (PLA)

In situ PLA was performed on HeLa cells grown on circular cover slips using the Duolink Detection Reagents Red (DUO92008, Sigma) according to the manufacturer’s instructions. The HeLa cells were fixed with 4% paraformaldehyde, permeabilized with 0.5% Triton X-100, and blocked with manufacturer-supplied Duolink Blocking Solution. Primary antibodies containing rabbit anti-VPS35 (ab226180, Abcam; 1:100 dilution) and mouse anti-Ku80 (66546-1-Ig, Proteintech Group; 1:100 dilution) were applied in a humidified chamber at 4℃ overnight. Following twice washes with Wash Buffer A (0.01 M Tris, 0.15 M NaCl and 0.05% Tween 20, pH7.4), the cells were incubated with Duolink anti-mouse PLA probe MINUS (DUO92004, Sigma) and Duolink anti-rabbit PLA probe PLUS (DUO92002, Sigma) in a humidity chamber for 1 h at 37℃. Subsequent enzymatic reactions involved sequential incubation with ligase (30 min, 37°C) and polymerase (100 min, 37°C), each step preceded by dual washes with Wash Buffer (A) Post-amplification cleaning was performed through two cycles of 1×Wash Buffer B (0.2 M Tris and 0.1 M NaCl, pH7.5) and one cycle of 0.01×diluted Wash Buffer (B) Nuclear DNA staining was achieved via 5 min Hoechst 33342 incubation at room temperature prior to confocal imaging (Zeiss LSM-710 or Nikon AXR).

### DNA affinity precipitation assay

HEK 293T cells grown in 10 cm dishes were lysed on ice for 10 min using 800 µL of whole cell lysis buffer (25 mM of HEPES (pH 7.7), 300 mM NaCl, 1.5 mM MgCl_2_, 0.2 mM EDTA, 0.5% Triton, 10% glycerol, 2 mM Na_3_VO_4_, 2 mM DL-Dithiothreitol (DTT), 0.5 mM PMSF, and proteinase inhibitors). Subsequently, 350 µL of the cell lysates were incubated at 4℃ for 30 min with either a 572 bp duplex DNA or its biotinylated counterpart, both of which were generated through PCR amplification of plasmid pG68. This was followed by a 2 h incubation at 4℃ with 20 µL of Streptavidin magnetic beads. The resulting protein-DNA-biotin-Streptavidin-bead complexes were collected using magnetic stand, washed five times with cold phosphate buffered saline (PBS), and then separated by 8% SDS-PAGE. The separated proteins were analyzed by Western blotting. All primers are listed in Table S1.

### Laser micro-irradiation

HEK-293T cells cultured in 24-well glass-bottom plates were transiently transfected with YFP-fusion constructs and/or mCherry-Ku70, followed by at least 24 h incubation in BrdU-supplemented medium (20 µM) prior to live-cell imaging. Immediately before laser micro-irradiation, culture medium was replaced with phenol-red-free medium to minimize background fluorescence during imaging. Targeted DNA damage induction was achieved through spatially controlled 405 nm laser exposure (70% maximal output, 10-pixel linear stripe pattern) using the FRAP module of Zeiss ZEN software. For DNA damage induction followed by immunofluorescence staining, cells were pretreated with 10 µg/mL Hoechst 33342 for 10 min instead of BrdU labeling, followed by laser micro-irradiation (405 nm laser at 92% output) under identical spatial parameters. Post-irradiation fixation (3-5 min after laser exposure) was performed using 4% paraformaldehyde for subsequent immunostaining procedures. Fluorescence quantification at DNA damage sites was performed through ImageJ. For individual cellular analysis, the fluorescence intensity values from laser-induced damage tracks were subjected to ratio-metric normalization against paired undamaged control regions, generating cell-specific relative fluorescence intensity.

### Chromatin fractionation assay

Cells grown on culture dishes were placed on ice and washed twice with ice-cold PBS, followed by collection using a cell scraper in 1 mL PBS. The cell suspension was centrifuged at 3,000 rpm for 2 min at 4°C, after which the supernatant was discarded. The pellet was resuspended in 600 µL ice-cold cytoskeleton buffer (20 mM HEPES pH7.8, 10 mM KCl, 2 mM EDTA, 300 mM sucrose, 0.5% Triton X-100, supplemented with protease inhibitor cocktail and 1 mM PMSF) by gentle pipetting, followed by 10 min incubation on ice for lysis. After centrifugation at 3,000 rpm for 3 min at 4°C, the supernatant containing soluble proteins was collected and the process was repeated with fresh cytoskeleton buffer for complete extraction. The final pellet was resuspended in 200 µL of chilled nuclear lysis buffer (10 mM HEPES pH7.8, 2 mM EDTA, 1 mM DTT) and subjected to sonication using the following parameters: 4 min total duration with 10% amplitude, 10 s pulse-on and 50 s pulse-off intervals, maintaining samples on ice throughout with the probe tip positioned at tube bottom. The sonicated lysate was centrifuged at 12,000 rpm for 20 min, after which the chromatin-containing supernatant was collected for subsequent western blot analysis.

### NHEJ assay and HR assay

NHEJ assay and HR assay were performed as we previously detailed (Zhang et al. 2024b). Cells seeded in 6-well plates were co-transfected with either 1 µg EJ5-GFP (for NHEJ) or 1 µg DR-GFP (for HR) reporter plasmid alongside 1 µg pCBASceI and 1 µg PCI2-HA-mCherry plasmids. Forty-eight hours after transfection, flow cytometry analysis was performed to detect GFP-positive (indicating successful NHEJ or HR repair) and mCherry-positive (transfection control) cells using a FACS Calibur flow cytometer (BD Biosciences) and FlowJo software. Repair efficiency was calculated as the percentage of GFP-positive cells normalized to mCherry-positive populations within each experimental condition, with final values expressed relative to control groups set as 100% repair capacity.

### Cell cycle analysis

Cells cultured in 6-well plates for 24 h were harvested by trypsinization, washed twice with ice-cold PBS, and stained with 0.5 mL PI solution (50 µg/mL propidium iodide, 0.1% Triton X-100, 0.1% sodium citrate, pH7.6) for 15 min at room temperature in the dark. Flow cytometry analysis was performed using a FACS Calibur flow cytometer (BD Biosciences) with data processed through FlowJo software.

### Statistical analysis

Statistical analysis was performed with GraphPad Prism 8.0 software. The unpaired, two-tailed Student’s t-test was used for comparisons between two groups, and one-way Analysis of Variance (ANOVA), followed by Dunnett’s multiple comparisons test, was employed for assessing differences among multiple groups. *p* < 0.05 was considered significant. *, *p* < 0.05, **, *p* < 0.01, ***, *p* < 0.001, ****, *p* < 0.0001; ns, not significant.

## Results

### VPS35 suppresses DNA repair

**Fig. 1:**
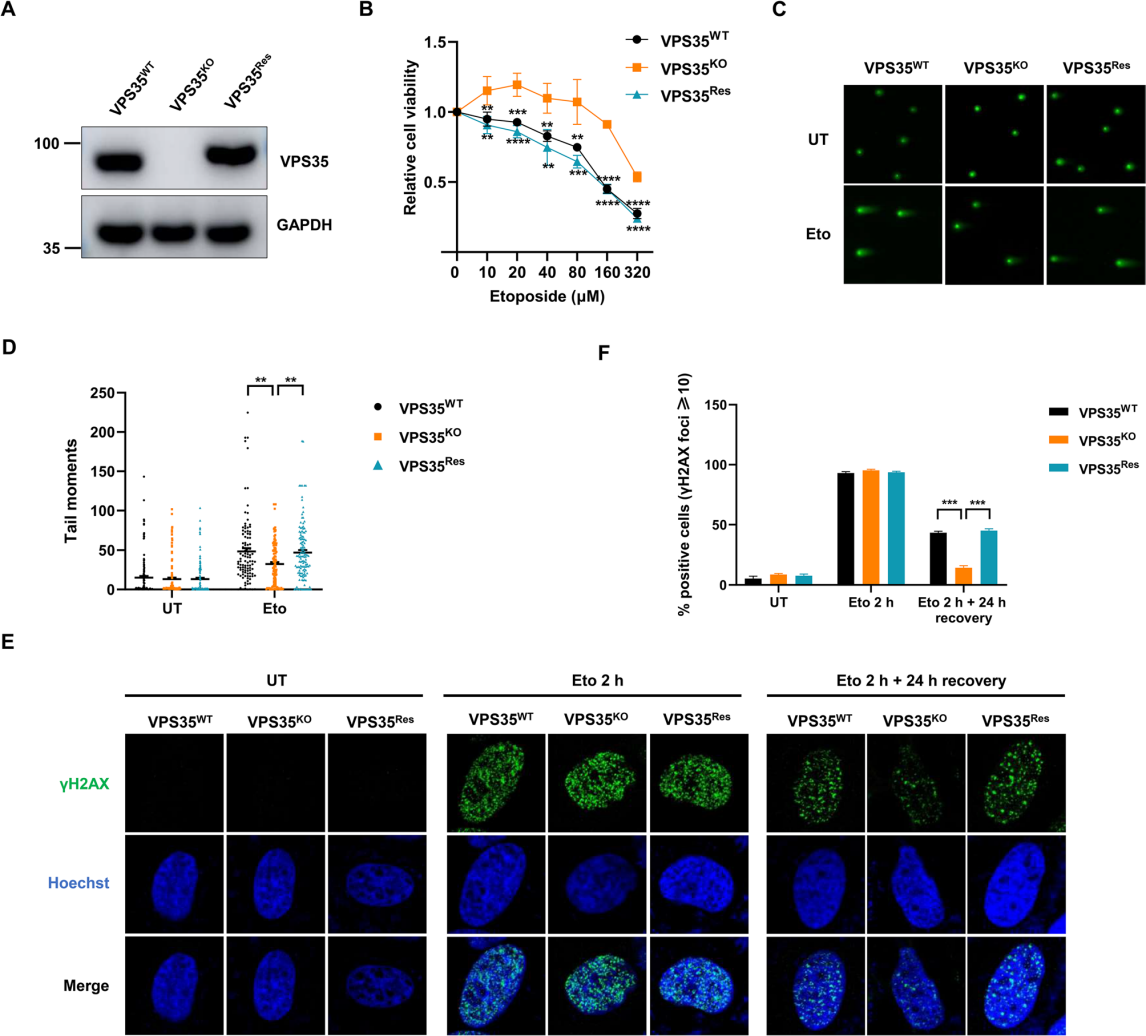
Loss of VPS35 promotes DNA DSB repair. **A** VPS35 expression in VPS35^WT^, VPS35^KO^ and VPS35^Res^ HeLa cells was measured by immunoblot. **B** Cell viability of VPS35^WT^, VPS35^KO^ and VPS35^Res^ HeLa cells treated with indicated concentration of etoposide for 48 h were measured with a CCK8 assay. Data are presented as mean ± SD. **, *p* < 0.01; ***, *p* < 0.001; ****, *p* < 0.0001. **C** VPS35^WT^, VPS35^KO^ and VPS35^Res^ HeLa cells treated with 100 µM etoposide for 60 min were allowed to recover for 30 min. Neutral Comet assay was performed to assess the DSB repair capacity. **D** Tail moments were quantified across three independent biological replicates, with 105–159 cells analyzed per experimental condition. Data are presented as mean ± SEM. **, *p* < 0.01. **E** Representative γH2AX foci of VPS35^WT^, VPS35^KO^ and VPS35^Res^ HeLa cells after etoposide treatment. Cells treated with 100 µM etoposide for 2 h were allowed to recover for 0-24 h under normal growth conditions, and then γH2AX foci were assessed. **F** The proportion of cells harboring > 10 γH2AX foci relative to the total cell population was quantified across three biological replicates, presented as mean ± SD through analysis of ≥ 250 randomly selected cells per condition. ***, *p* < 0.001

We have previously reported WASH and FAM21 promote DNA repair by interacting with Ku protein (Wang et al. 2022; Wang et al. 2024). In light of the established interplay between FAM21 and VPS35, we were intrigued to delve into the possible DDR roles of VPS35. To our surprise, knockout (KO) of VPS35 by CRISPR/Cas9 desensitized HeLa cells to etoposide, a topoisomerase II inhibitor that induces DSBs (Fig. 1A-B). To rule out off-target effects of the VPS35 guide RNA (gRNA), we re-expressed wild-type (WT) VPS35 in VPS35^KO^ cells. This restoration of WT VPS35 rescued the DNA damage insensitivity conferred by VPS35 deficiency (Fig. 1A-B), indicating VPS35 sensitizes cells to DNA DSBs. To further examine whether VPS35 plays a role in DSB repair, we performed a neutral Comet assay on VPS35^WT^ + Vector (hereafter referred to as VPS35^WT^), VPS35^KO^ + Vector (hereafter referred to as VPS35^KO^) and VPS35^KO^ + VPS35^Rescue^ (hereafter referred to as VPS35^Res^) cells. The VPS35^KO^ cells showed a reduced number of unrepaired DSBs compared to VPS35^WT^ cells, after 30 min of recovery post etoposide treatment (Fig. 1C-D). Restoration of VPS35 expression led to an increase in unrepaired DSBs compared to VPS35^KO^ cells, indicating VPS35 interferes with DNA repair (Fig. 1C-D). Next, we examined γH2AX foci resolution in VPS35^WT^, VPS35^KO^ and VPS35^Res^ cells exposed to etoposide. In contrast to VPS35^WT^ cells, VPS35^KO^ cells exhibited a less sustained γH2AX foci pattern, after 24 h of recovery post etoposide treatment (Fig. 1E-F). Notably, this phenotype was rescued by VPS35 re-expression (Fig. 1E-F). We also extended these findings using bleomycin, a radiomimetic compound inducing complex DSBs. Consistent with our etoposide results, VPS35^KO^ cells exhibited enhanced survival and more efficient DSB repair compared to VPS35^WT^ controls, while VPS35^Res^ cells largely phenocopied the VPS35^WT^ cells response (Fig. S1). Collectively, these results strongly suggest that VPS35 suppresses DNA damage repair. To examine the role of nuclear VPS35 in modulating DNA repair, we stably overexpressed the NLS-tagged VPS35 (NLS.VPS35) or a control vector in HEK-293T cells using a lentivirus-mediated stable overexpression system (Fig. S2A). The overexpression of NLS.VPS35 nearly abolished endogenous VPS35 expression, likely due to a dominant-negative effect. The resultant NLS.VPS35^OE^ cells exhibited predominant nuclear expression of VPS35 (Fig. S2B). Importantly, NLS.VPS35^OE^ cells showed pronounced hypersensitivity to both etoposide and bleomycin (Fig. S2C-D). Neutral Comet assays confirmed impaired DSB repair in NLS.VPS35^OE^ cells, with increased sustained DSBs retention after 30 min of recovery post bleomycin or etoposide treatment compared to Vector^OE^ cells (Fig. S2E-H). These results provide evidence that nuclear-localized VPS35 actively inhibits DNA repair.

### VPS35 interacts with Ku protein

**Fig. 2:**
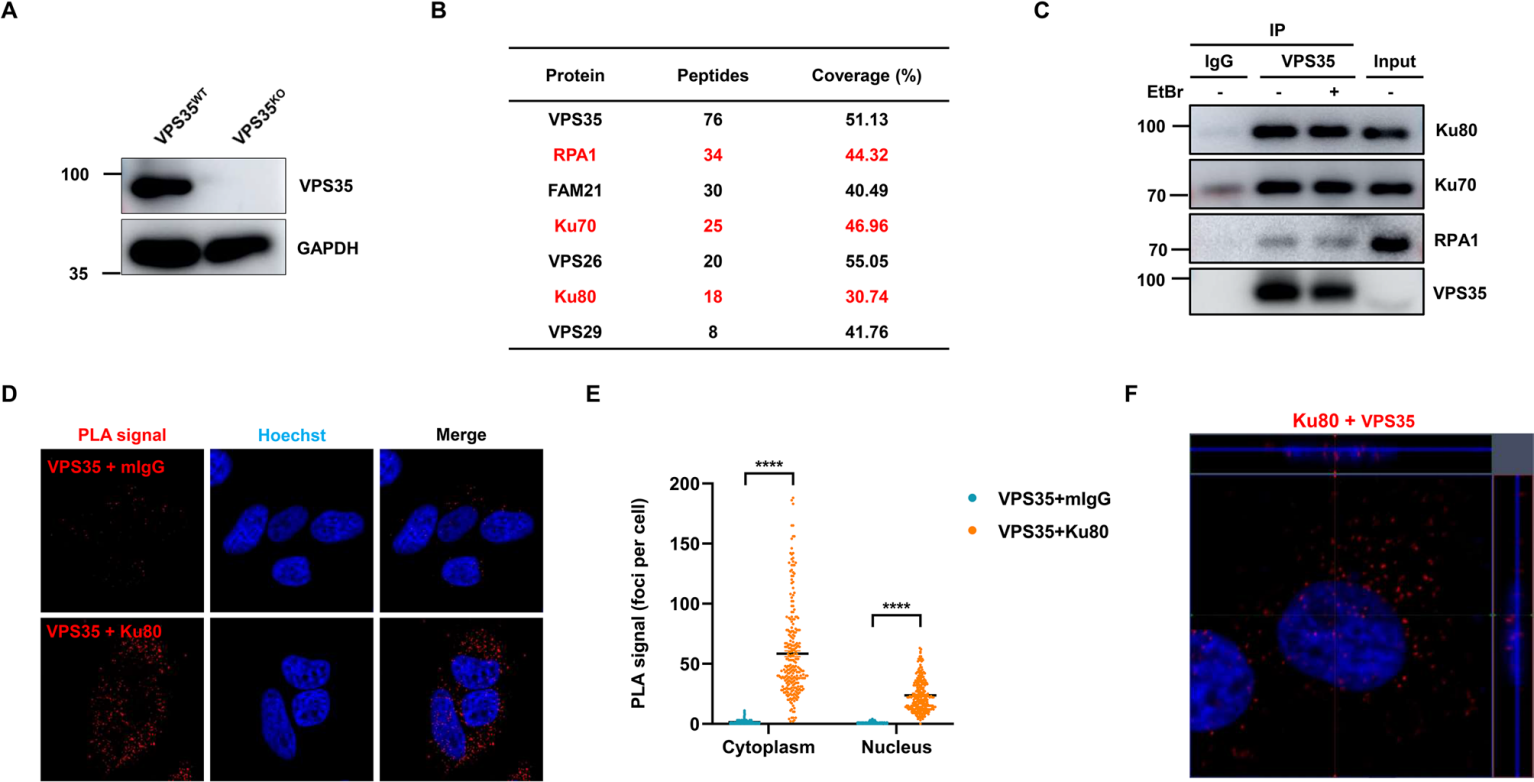
VPS35 interacts with Ku protein. **A** VPS35 expression in VPS35^WT^ and VPS35^KO^ HEK-293T cells was measured by immunoblot. **B** The cell lysates of VPS35^KO^ HEK-293T cells transfected with PCI2.Flag.VPS35 were subjected to anti-Flag affinity purification and analyzed by mass spectrometry. **C** The interactions between endogenous VPS35 and Ku protein were determined by co-immunoprecipitation assay. HEK-293T cell lysates were immunoprecipitated with anti-VPS35 or rabbit IgG in the absence or presence of EtBr (0.2 mg/mL) followed by SDS-PAGE and immunoblotting. **D** The interactions between VPS35 and Ku80 were assessed by PLA with the indicated pairs of primary antibodies in HeLa cells. Co-staining VPS35 with mouse IgG were used as negative control. **E** PLA signal foci numbers per nucleus in 256 cells of each group from three independent biological replicates were calculated and presented as mean ± SEM. ****, *p* < 0.0001. **F** Example of confocal z-stack images and orthogonal views for the PLA signals between VPS35 and Ku80

To search for potential DDR partners of VPS35, we first constructed VPS35 knockout (VPS35^KO^) HEK-293T cells using the CRISPR/Cas9 system (Fig. 2A). We then overexpressed Flag-tagged VPS35 in these VPS35^KO^ HEK-293T cells and purified the Flag.VPS35 using anti-Flag antibody. The isolated Flag.VPS35 and its associated proteins were subjected to mass spectrometry analysis (Table S2). This analysis revealed several established VPS35-associated proteins, such as FAM21, VPS26 and VPS29. Notably, the Ku70/Ku80 heterodimer, known as the initiator of NHEJ pathway, and replication protein A-1 (RPA1), a core HR component, were also identified (Fig. 2B). To confirm these interactions, we performed coimmunoprecipitation (Co-IP) assay with antibody against VPS35. As shown in Fig. 2C, endogenous VPS35 clearly interacts with Ku70 and Ku80 in cells, while VPS35 only slightly interacts with RPA1. Moreover, these interactions appeared to be largely independent of DNA, as ethidium bromide (EtBr) treatment, which disrupts the majority of protein-DNA interactions, had limited impact on the co-immunoprecipitation. To further define the subcellular localization of the interaction between VPS35 and Ku, we performed an in situ proximity ligation assay (PLA), which enables the visual monitoring of protein-protein interactions in close proximity within individual cells. Co-staining of VPS35 with Ku80 yielded pronounced PLA signals in both the nucleus and the cytoplasm in contrast to control cells (Fig. 2D-E and Fig. S3). Cytoplasmic interactions displayed higher frequency compared to nuclear interactions (Fig. 2E), which corresponds with subcellular fractionation assays showing VPS35 was predominantly localized in the cytoplasm but also present in the nucleus (Fig. S4). Notably, the nuclear PLA signals suggest that the interaction between VPS35 and Ku occurs, at least partially, within the nuclear compartment (Fig. 2F).

### VPS35 localizes to DNA damage sites independent of Ku protein

**Fig. 3:**
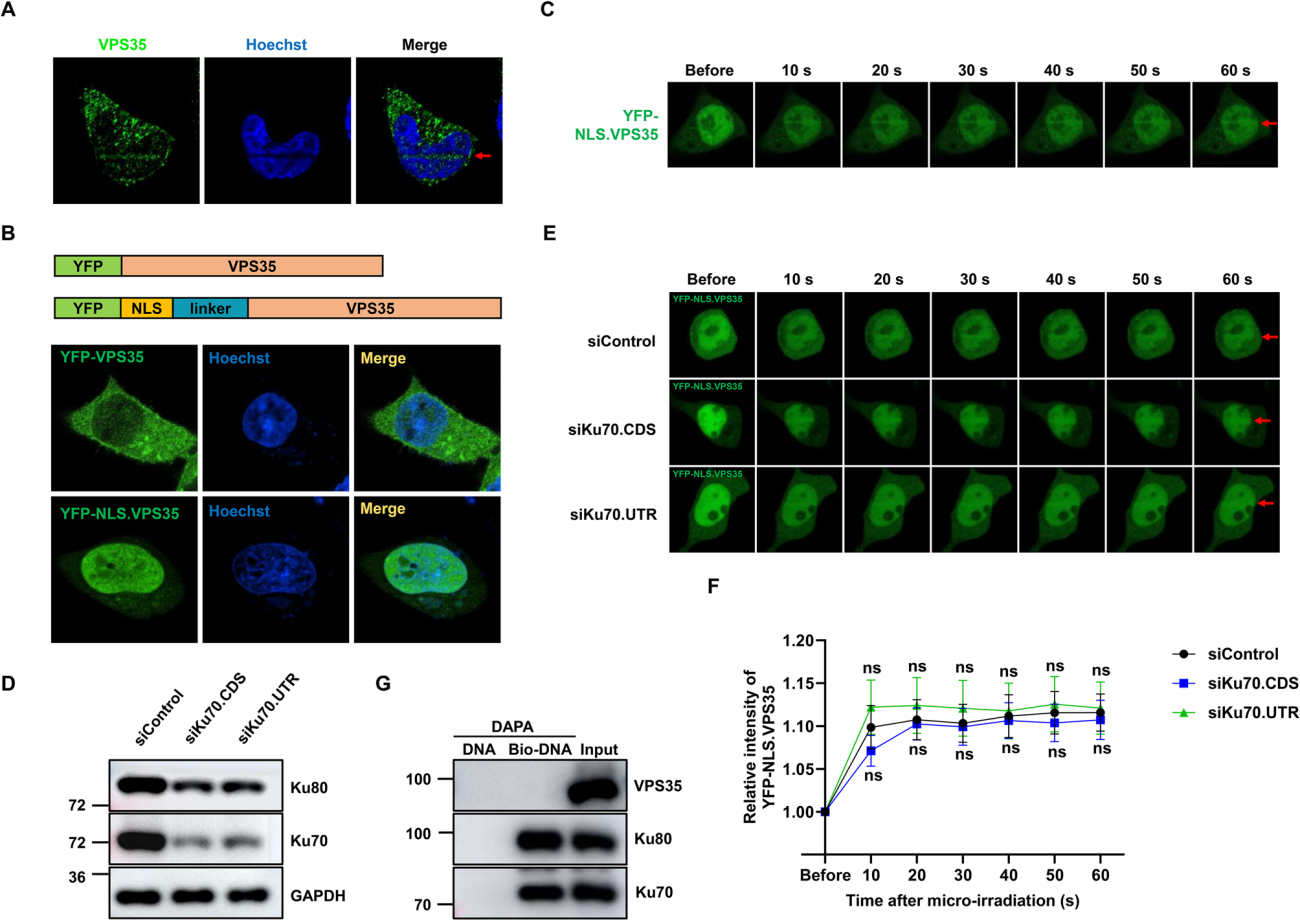
VPS35 localizes to DNA damage sites independent of Ku protein. **A** Laser micro-irradiation was used to induce DNA damage in HeLa cells. Three minutes after micro-irradiation, cells were fixed and subjected to immunofluorescence. **B** HeLa cells were transfected with YFP.VPS35 and YFP-NLS.VPS35 for YFP fluorescence imaging. **C** Laser micro-irradiation was used to induce DNA damage in a line pattern in HEK-293T cells transfected with YFP-NLS.VPS35. **D** Expression levels of Ku70 and Ku80 in HEK-293T cells transfected with either control siRNA (siControl) or siRNAs targeting Ku70 were examined by immunoblot. **E** Recruitment of nuclear VPS35 to laser-generated DNA damage sites is measured in siKu70 and siControl cells. **F** Quantification of nuclear VPS35 recruitment to DNA damage tracks in control and Ku70-depleted cells. The relocation kinetics of nuclear VPS35 to DNA damage sites was monitored in a time course as indicated. Laser micro-irradiation experiments were conducted across three independent biological replicates (≥ 20 cells), with the results presented as mean ± SEM. ns, not significant. **G** DAPA were carried out using biotin labeled DNA and HEK-293T cell lysates. DNA-bound Ku70, Ku80 and VPS35 were detected by immunoblot

The interaction between VPS35 and Ku led us to investigate if VPS35 is present at DNA damage sites. Laser micro-irradiation was used to induce localized DNA damage, followed by analysis through immunofluorescent staining. We found that endogenous VPS35 was modestly recruited to the DSBs induced by laser micro-irradiation (Fig. 3A). To gain a more nuanced understanding of the kinetics of VPS35 recruitment to DNA lesions, we constructed a plasmid expressing nuclear VPS35, designated as YFP-NLS.VPS35, by inserting the SV40 nuclear localization sequence (NLS) between YFP tag and the VPS35 coding sequence (CDS). The subcellular localization of YFP-NLS.VPS35 was predominantly nuclear (Fig. 3B), with a fraction of YFP-NLS.VPS35 promptly recruited to laser-induced damage tracks following micro-irradiation within a 10-second timeframe (Fig. 3C). To further determine whether the VPS35 recruitment to DSBs is dependent on Ku protein, two siRNA targeting Ku70 were used to suppress Ku70 expression, leading to concomitant reduction in Ku80 levels due to their mutual stability dependence (Fig. 3D). Unexpectedly, depletion of Ku protein did not obviously impact the recruitment of VPS35 to DSBs (Fig. 3E-F), suggesting that the recruitment of VPS35 is not contingent upon Ku protein presence. In addition, in the DNA affinity precipitation assay, VPS35 was not detected in the biotin-labeled DNA pull-down precipitates, indicating that VPS35 does not interact with broken DNA (Fig. 3G). These findings collectively suggest that although VPS35 may accumulate in small quantities around sites of DNA damage, it does not interact with DNA, and this accumulation is not dependent on Ku protein.

### Nuclear VPS35 suppresses the recruitment of Ku70 to DNA damage sites

**Fig. 4:**
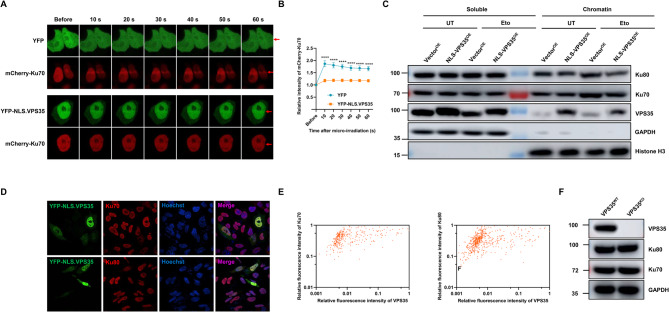
Nuclear VPS35 inhibits Ku70 recruitment to DNA damage sites. **A** Laser micro-irradiation was used to induce DNA damage in a line pattern in HEK-293T cells co-transfected with mcherry-Ku70 and pCMS3.HA-YFP plasmids (Control) or mcherry-Ku70 and YFP-NLS.VPS35 plasmids. **B** Quantification of nuclear Ku recruitment to DNA damage tracks in control and nuclear VPS35 overexpression cells. The relocation kinetics of Ku70 to DNA damage sites was monitored in a time course as indicated. Laser micro-irradiation experiments were conducted across three independent biological replicates (≥ 20 cells), with the results are presented as mean ± SEM. ****, *p* < 0.0001. **C** Vector^OE^ and NLS.VPS35^OE^ HEK-293T cells treated with or without 100 µM etoposide for 60 min were subjected to chromatin fractionation assay followed by SDS-PAGE and immunoblotting. **D** Ku70 or Ku80 expression in HeLa cells transfected with YFP-NLS.VPS35 was measured by immunofluorescence. **E** Relative fluorescence intensity of Ku70/Ku80 and YFP-NLS.VPS35 per cell for 337-598 cells of each group were calculated. **F** Ku70, Ku80 and VPS35 expression in VPS35^WT^ and VPS35^KO^ HEK-293T cells was measured by immunoblot

The next question is how VPS35 inhibits DNA repair. We have previously reported WASH and FAM21 localize to DNA damage sites and involve in DDR (Wang et al. 2022; Wang et al. 2024). Given that VPS35 interacts with both WASH/FAM21 and Ku proteins, we initially hypothesized that VPS35 might influence the recruitment of WASH/FAM21 to DNA damage sites. To test this hypothesis, we transfected VPS35^WT^ and VPS35^KO^ HEK-293T cells with either the YFP-NLS.WASH or the nuclear-targeted FAM21 truncation construct YFP-FAM21.Δ219N, a well-characterized tool for monitoring FAM21 recruitment dynamics (Wang et al. 2024). The recruitment of YFP-NLS.WASH and YFP-FAM21.Δ219N to DNA damage sites were analyzed using laser micro-irradiation. Contrary to our expectations, no significant difference in WASH and FAM21 recruitment was observed between VPS35^WT^ and VPS35^KO^ cells (Fig. S5A-D). Furthermore, Co-IP assay corroborated these findings, demonstrating that VPS35 depletion did not impact the interaction of WASH with Ku protein (Fig. S5E). Collectively, these results suggest that VPS35 does not directly mediate the recruitment of WASH and FAM21 to DNA damage sites, based on the current evidence. The recognition of Ku to the damaged DNA ends is critical to the initiation of NHEJ. Given the interaction between VPS35 and the Ku protein, we asked whether the nuclear VPS35 influences the recruitment of Ku to DNA damage sites. To this end, we co-transfected HEK-293T cells with YFP-NLS.VPS35 and mcherry-Ku70. In contrast to control cells co-transfected with mcherry-Ku70 and an empty pCMS3.H1p/HA.YFP plasmid, overexpression of nuclear VPS35 significantly impeded the recruitment of Ku70 to the laser induced DNA damage tracks (Fig. 4A-B). This result suggests nuclear VPS35 functions as a suppressor of Ku70 recruitment to DNA damage sites. This finding was further corroborated by chromatin fractionation assays showing that while etoposide treatment enhanced Ku70/Ku80 chromatin association in control cells, NLS-VPS35 overexpression attenuated this damage-induced recruitment (Fig. 4C), consistent with our proposed model that VPS35 disrupts Ku protein recruitment to DSBs. Interestingly, while NLS-VPS35 clearly disrupted Ku recruitment to chromatin-associated DSBs, DNA affinity precipitation assays revealed no significant difference in Ku70/Ku80 binding to naked biotinylated DNA between NLS-VPS35^OE^ and Vector^OE^ cells (Fig. S6), suggesting the inhibitory function of VPS35 likely depends on chromatin context or spatial regulation rather than direct competition for DNA binding. To exclude potential effects on Ku protein levels, we confirmed through immunofluorescence in YFP-NLS.VPS35-transfected HeLa cells and immunoblotting in VPS35^WT^ versus VPS35^KO^ cells that neither VPS35 overexpression nor deletion alters Ku70/Ku80 expression (Fig. 4D-F), demonstrating that the observed changes in Ku recruitment and repair efficiency reflect functional regulation rather than alterations in Ku protein abundance or stability.

### VPS35 restrains the activation of DNA-PKcs

**Fig. 5:**
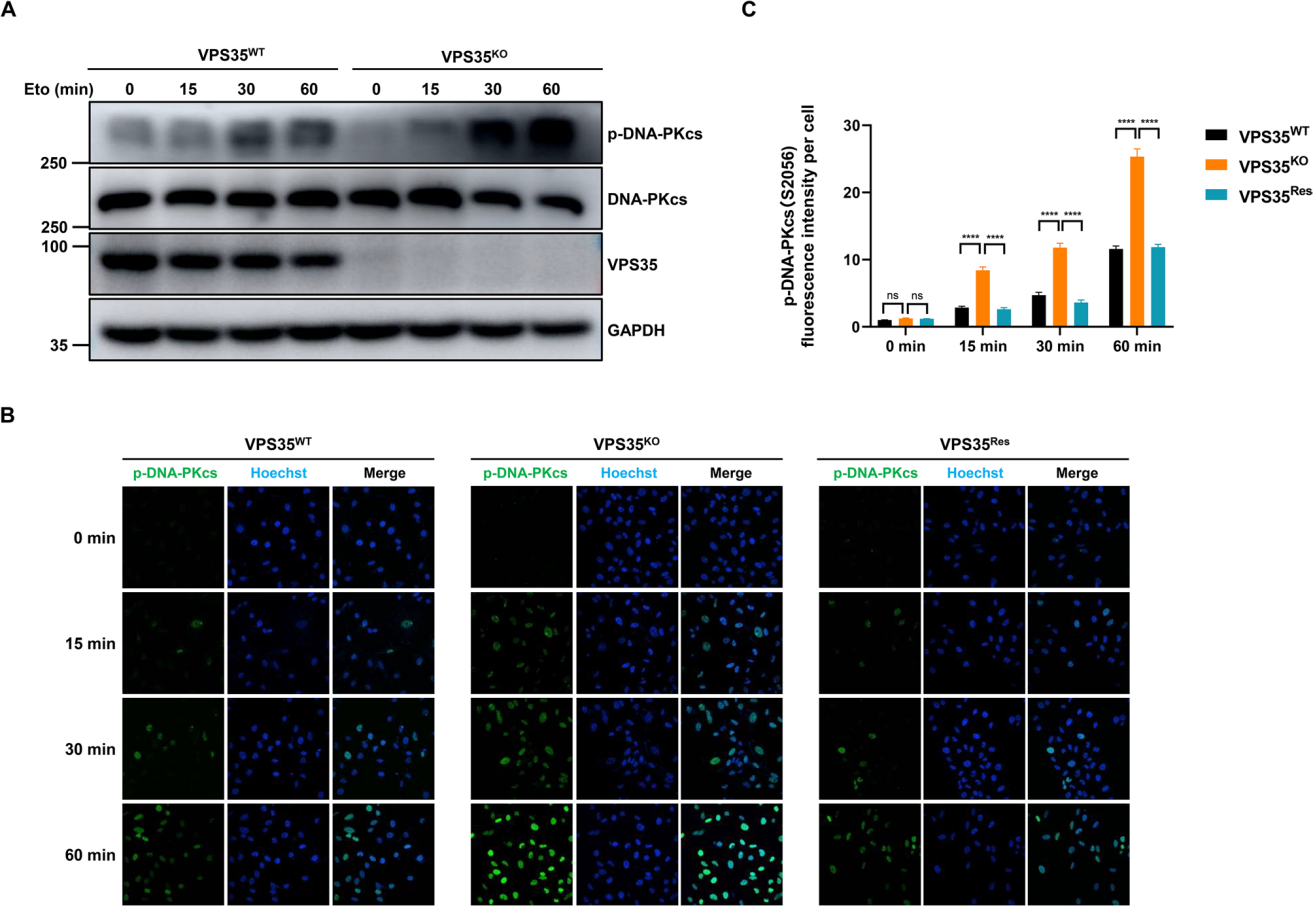
VPS35 inhibits DNA-PKcs activation. **A** VPS35^WT^ and VPS35^KO^ HeLa cells were treated with 100 µM etoposide for the indicated time. Whole cell lysates were obtained and immunoblotted with the indicated antibodies. **B** Representative phosphorylation of DNA-PKcs (S2056) of VPS35^WT^, VPS35^KO^ and VPS35^Res^ HeLa cells after etoposide treatment. VPS35^WT^ and VPS35^KO^ HeLa cells were treated with 100 µM etoposide for 0, 15, 30-60 min. Then fluorescence intensity of p-DNA-PKcs (S2056) were assessed. **C** Fluorescence intensity of p-DNA-PKcs (S2056) per cell for 203-254 cells in each group were calculated across three biological replicates and presented as mean ± SEM. ****, *p* < 0.0001; ns, not significant

The activation of DNA-PKcs is required for effective DSB repair and is dependent on the binding of Ku protein to DNA damage sites. The impact of VPS35 on Ku70 recruitment to DSBs prompted us to examine its potential regulation of DNA-PKcs activation. The autophosphorylation of DNA-PKcs at serine 2056 is a well-documented indicator of its activation status. As shown in Fig. 5A, etoposide induced autophosphorylation of DNA-PKcs at S2056 was significantly increased in the VPS35^KO^ cells, suggesting a suppressive role of VPS35 in DNA-PKcs activation. This finding was further supported by immunofluorescence studies, which revealed heightened levels of etoposide induced DNA-PKcs S2056 phosphorylation in VPS35^KO^ cells compared to VPS35^WT^ cells, unequivocally confirming the restraining effect of VPS35 on DNA-PKcs activation (Fig. 5B-C).

### Nuclear VPS35 inhibits NHEJ

**Fig. 6:**
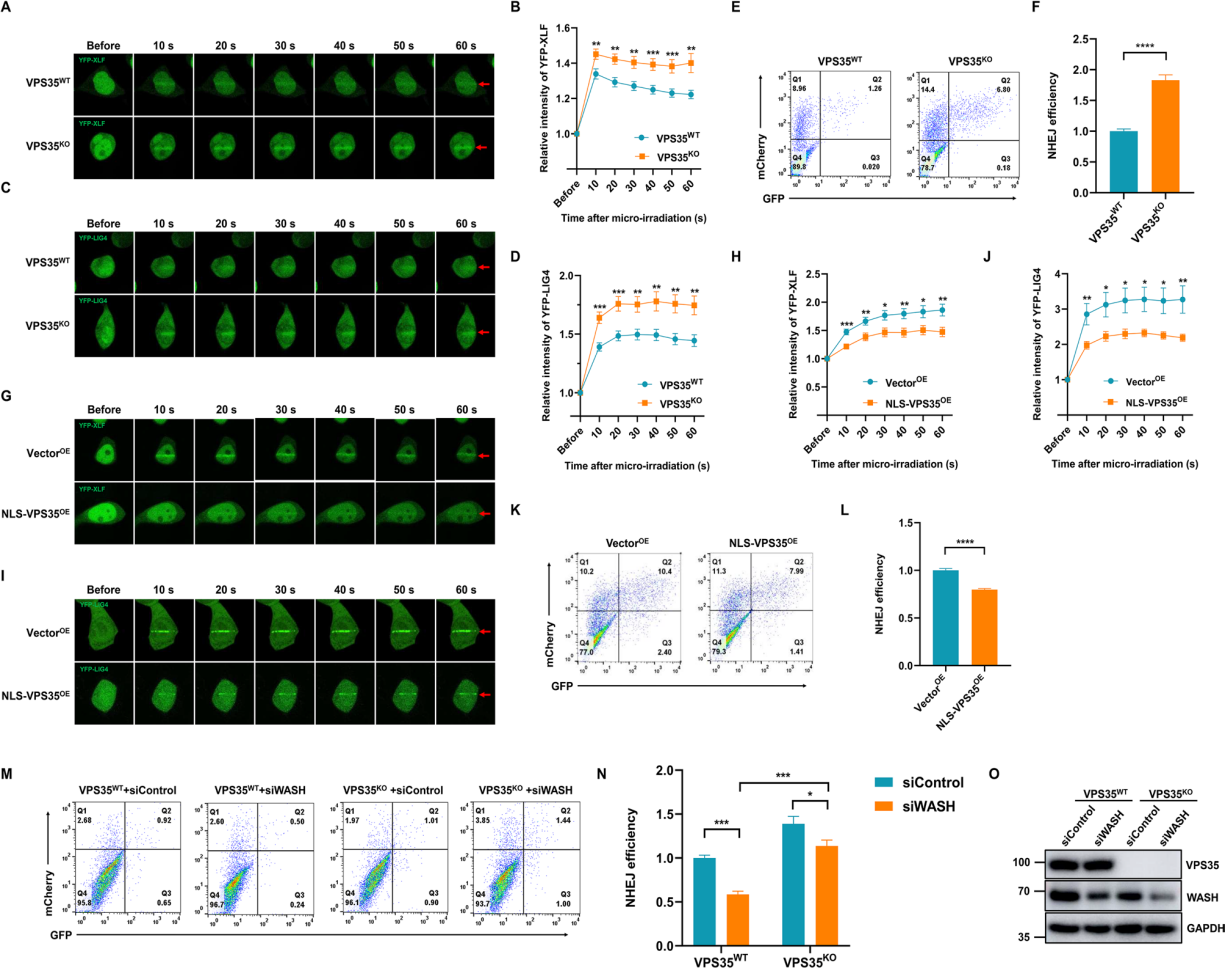
Nuclear VPS35 inhibits NHEJ pathway. **A** Laser micro-irradiation was used to induce DNA damage in a line pattern in VPS35^WT^ and VPS35^KO^ HEK-293T cells transfected with YFP-XLF. **B** Quantification of XLF recruitment to DNA damage tracks in VPS35^WT^ and VPS35^KO^ HEK-293T cells. Laser micro-irradiation experiments were conducted across three independent biological replicates (≥ 20 cells), with the results are presented as mean ± SEM. **, *p* < 0.01; ***, *p* < 0.001. **C** Laser micro-irradiation was used to induce DNA damage in a line pattern in VPS35^WT^ and VPS35^KO^ HEK-293T cells transfected with YFP-LIG4. **D** Quantification of DNA-Ligase 4 recruitment to DNA damage tracks in VPS35^WT^ and VPS35^KO^ HEK-293T cells. Laser micro-irradiation experiments were conducted across three independent biological replicates (≥ 20 cells), with the results are presented as mean ± SEM. **, *p* < 0.01; ***, *p* < 0.001. **E** VPS35^WT^ and VPS35^KO^ HEK-293T cells were subjected to NHEJ assay. **F** NHEJ efficiency was calculated by comparing the number of GFP-positive cells with the number of mCherry-positive cells. Data were presented as mean ± SD. ****, *p* < 0.0001. **G** Laser micro-irradiation was used to induce DNA damage in a line pattern in NLS.VPS35^OE^ and Vector^OE^ HEK-293T cells transfected with YFP-XLF. **H** Quantification of XLF recruitment to DNA damage tracks in NLS.VPS35^OE^ and Vector^OE^ HEK-293T cells. Laser micro-irradiation experiments were conducted across three independent biological replicates (≥ 20 cells), with the results are presented as mean ± SEM. *, *p* < 0.05, **, *p* < 0.01; ***, *p* < 0.001. **I** Laser micro-irradiation was used to induce DNA damage in a line pattern in NLS.VPS35^OE^ and Vector^OE^ HEK-293T cells transfected with YFP-LIG4. **J** Quantification of DNA-Ligase 4 recruitment to DNA damage tracks in NLS.VPS35^OE^ and Vector^OE^ HEK-293T cells. Laser micro-irradiation experiments were conducted across three independent biological replicates (≥ 20 cells), with the results are presented as mean ± SEM. *, *p* < 0.05, **, *p* < 0.01. **K** NLS.VPS35^OE^ and Vector^OE^ HEK-293T cells were subjected to NHEJ assay. **L** NHEJ efficiency was calculated by comparing the number of GFP-positive cells with the number of mCherry-positive cells. Data were presented as mean ± SD. ****, *p* < 0.0001. **M** WASH was depleted by siRNA-mediated gene silencing in VPS35^WT^ and VPS35^KO^ HEK-293T cells and then cells were subjected to NHEJ assay. **N** NHEJ efficiency was calculated by comparing the number of GFP-positive cells with the number of mCherry-positive cells. Data were presented as mean ± SD. *, *p* < 0.05, ***, *p* < 0.001. **O** VPS35 and WASH expression in VPS35^WT^ and VPS35^KO^ HEK-293T cells transfected with siControl or siWASH was measured by immunoblot

The catalytic activity of DNA-PKcs is essential for the recruitment of DNA DDR proteins and the efficiency of NHEJ (Lu et al. 2019). Given the role of VPS35 in regulating DNA-PKcs activation, we delved into its potential influence on the recruitment of downstream NHEJ factors and NHEJ efficiency. Consistent with its suppressive role in DNA-PKcs activation, VPS35 KO led to increased recruitment of YFP-tagged XLF and DNA-Ligase 4, suggesting VPS35 curtails the recruitment of the NHEJ machinery to DSBs (Fig. 6A-D). Furthermore, the efficiency of NHEJ in VPS35^WT^ and VPS35^KO^ HEK-293T cells was evaluated in vivo via a GFP reporter assay, a widely recognized method for assessing NHEJ efficiency (Zhang et al. 2024b). VPS35^KO^ cells exhibited a significant increase in NHEJ-mediated DSB repair compared to VPS35^WT^ cells (Fig. 6E-F). Moreover, overexpression of VPS35 in HEK293T cells inhibited NHEJ efficiency, further validating its functional role as an NHEJ suppressor (Fig. S7). Notably, overexpression of NLS-VPS35 led to reduced recruitment of XLF and DNA-Ligase 4 (Fig. 6G-J), accompanied by a decrease in NHEJ efficiency (Fig. 6K-L). Taken together, these cumulative findings incontrovertibly demonstrate that nuclear VPS35 acts as an inhibitor of the NHEJ pathway. To elucidate the mechanistic interplay between VPS35 and WASH/FAM21 in modulating NHEJ, we performed an epistatic relationship analysis by transfecting siWASH or siControl into both VPS35^WT^ and VPS35^KO^ HEK-293T cells. WASH depletion significantly reduced NHEJ efficiency in both VPS35^KO^ and VPS35^WT^ cells, while VPS35 knockout consistently enhanced NHEJ efficiency regardless of WASH expression (Fig. 6M-O). This non-epistatic relationship suggests that WASH and VPS35 regulate NHEJ through parallel, non-overlapping pathways rather than operating within a linear hierarchy, with their opposing functional impacts on repair efficiency arising from distinct molecular mechanisms. The observation that VPS35 suppresses NHEJ prompted us to investigate its potential role in the HR pathway. Using HR assays in VPS35^KO^ and NLS-VPS35^OE^ cells alongside their respective controls, we found that VPS35 knockout significantly enhanced HR efficiency (Fig. S8A-B), while NLS-VPS35 overexpression suppressed HR efficiency (Fig. S8C-D). These results indicate the function of VPS35 in HR is consistent with its inhibitory effect on NHEJ. The parallel enhancement or suppression of both NHEJ and HR in VPS35 mutants suggests that VPS35 acts as a global suppressor of DSB repair rather than a regulator of pathway choice. To investigate the mechanism underlying VPS35-mediated suppression of HR, we examined chromatin-bound RPA1, an HR factor we have shown interacts with VPS35 (Fig. 2B-C), in Vector^OE^ and VPS35^OE^ cells. Notably, overexpression of NLS-VPS35 did not alter chromatin-associated RPA1 levels following etoposide-induced DNA damage (Fig. S8E), suggesting that VPS35 does not impair HR efficiency through RPA1 regulation. These findings further indicate that VPS35 selectively attenuates NHEJ factor recruitment while permitting initial HR factor loading to chromatin, although it suppresses both NHEJ and HR efficiency. To rule out the possibility that changes in NHEJ and HR efficiency were partially affected by cell cycle alterations, we analyzed the cell cycle distribution of VPS35^KO^ and NLS-VPS35^OE^ cells along with their corresponding control cells. Both mutants exhibited profiles similar to their respective controls (Fig. S9), confirming that VPS35 does not significantly impact cell cycle progression. Therefore, the observed modulation of NHEJ and HR efficiencies can be unequivocally attributed to the direct regulatory role of VPS35 in DDR rather than secondary cell cycle effects.

### VPS35 dissociates from Ku protein in response to DNA damage

**Fig. 7:**
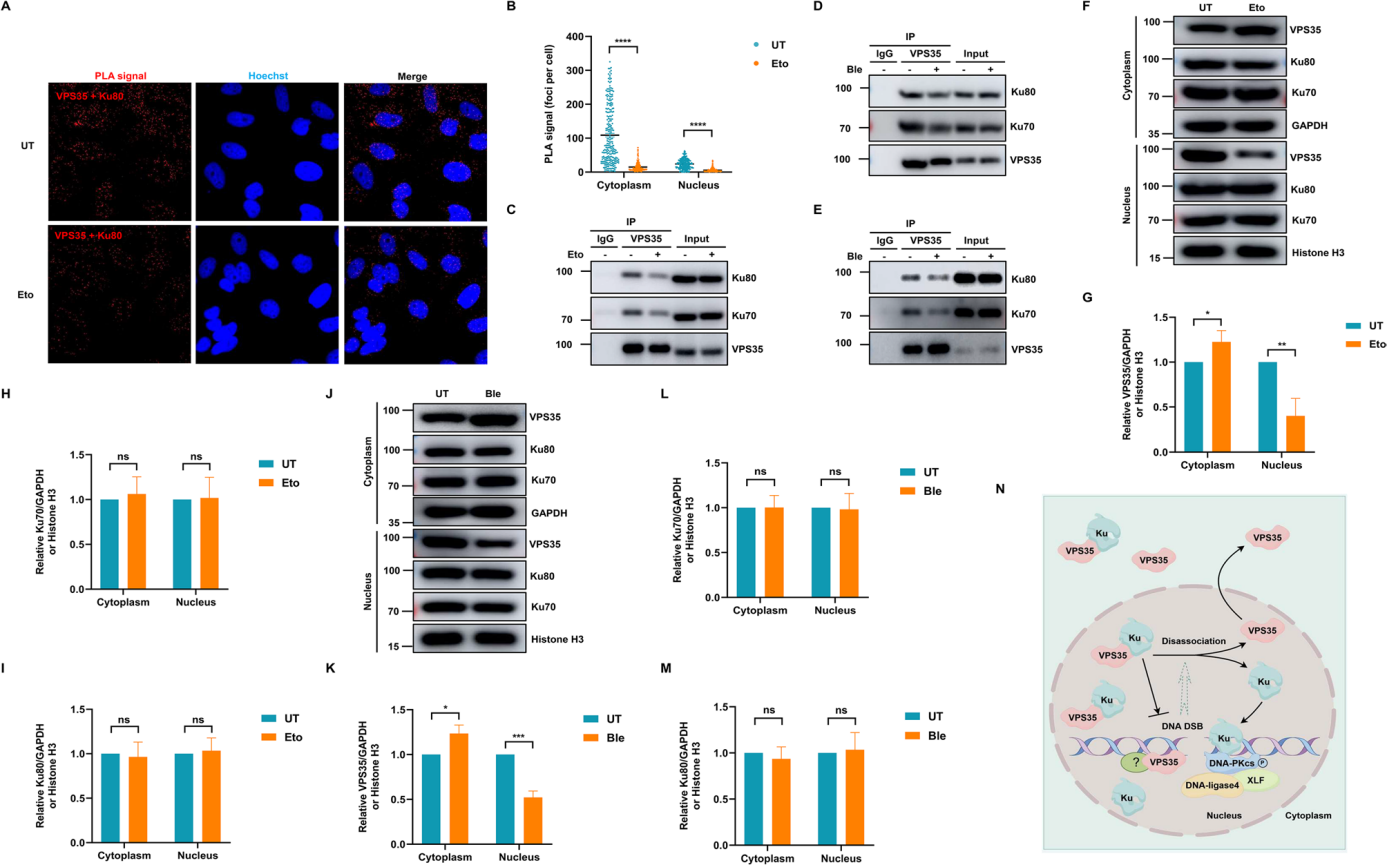
VPS35-Ku interaction is dissociated after DNA damage. **A** The interaction between VPS35 and Ku80 was assessed by PLA in HeLa cells with 100 µM etoposide treatment for 2 h or not. **B** PLA signal foci numbers per nucleus for 256 cells in each group were calculated across three independent biological replicates and presented as mean ± SEM. ****, *p* < 0.0001. **C** HEK-293T cells treated with or without 100 µM etoposide for 2 h were lysed and subjected to anti-VPS35 Co-IP assay followed by SDS-PAGE and immunoblotting. **D** HEK-293T cells treated with or without 200 µg/mL bleomycin for 2 h were lysed and subjected to anti-VPS35 Co-IP assay followed by SDS-PAGE and immunoblotting. **E** Serum-starved HEK-293T cells (24 h in serum-free medium) were treated with or without 200 µg/mL bleomycin for 2 h, followed by anti-VPS35 Co-IP, SDS-PAGE, and immunoblotting. **F** The cytoplasmic and nuclear extracts from HeLa cells with 100 µM etoposide treatment for 2 h or not were analyzed by immunoblot. GAPDH and Histone 3 serve as cytoplasmic and nuclear markers, respectively. **G** Quantification of VPS35 expression in cytoplasm and nucleus, normalized to GAPDH and Histone H3, respectively. Results were calculated across three independent biological replicates and presented as mean ± SD. *, *p* < 0.05; **, *p* < 0.01. **H** Quantification of Ku70 expression in cytoplasm and nucleus, normalized to GAPDH and Histone H3, respectively. Results were calculated across three independent biological replicates and presented as mean ± SD. ns, not significant. **I** Quantification of Ku80 expression in cytoplasm and nucleus, normalized to GAPDH and Histone H3, respectively. Results were calculated across three independent biological replicates and presented as mean ± SD. ns, not significant. **J** The cytoplasmic and nuclear extracts from HeLa cells with 200 µg/mL bleomycin treatment for 2 h or not were analyzed by immunoblot. GAPDH and Histone 3 serve as cytoplasmic and nuclear markers, respectively. **K** Quantification of VPS35 expression in cytoplasm and nucleus, normalized to GAPDH and Histone H3, respectively. Results were calculated across three independent biological replicates and presented as mean ± SD. *, *p* < 0.05; ***, *p* < 0.001. **L** Quantification of Ku70 expression in cytoplasm and nucleus, normalized to GAPDH and Histone H3, respectively. Results were calculated across three independent biological replicates and presented as mean ± SD. ns, not significant. **M** Quantification of Ku80 expression in cytoplasm and nucleus, normalized to GAPDH and Histone H3, respectively. Results were calculated across three independent biological replicates and presented as mean ± SD. ns, not significant. **N** A proposed model for the role of VPS35 in DNA repair. The figure was produced using Figdraw

In unperturbed cells, a discernible interaction between VPS35 and Ku protein was observed (Fig. 2), hinting at the potential physiological importance of this interplay. Subsequently, we studied the dynamics of this interaction in the context of DNA damage. A decrease in PLA dots of VPS35 and Ku80 was detected in cells treated with etoposide for 2 h (Fig. 7A-B), demonstrating damage-triggered complex disassembly. This dissociation was biochemically validated through Co-IP assays showing interaction decreases under etoposide or bleomycin treatments (Fig. 7C-D), with the dissociation phenotype persisting in G0/G1-synchronized cells (Fig. 7E). The subcellular fractionation analysis further confirmed the diminished presence of VPS35 in the nuclear fraction upon DNA damage induced by etoposide or bleomycin, while Ku protein maintained stable nuclear-cytoplasmic distribution, thus suggesting a translocation of VPS35 to the cytoplasm (Fig. 7F-M). This relocation may represent a regulatory mechanism that liberates Ku proteins, enabling them to perform their crucial DNA repair functions (Fig. 7N).

## Discussion

Despite the well-documented dual localization of VPS35 in both the cytoplasm and nucleus, its precise nuclear role has remained elusive (Deng et al. 2015). Our research now sheds light on this mystery, revealing a novel suppressive function of VPS35 in DNA repair efficiency. Specifically, we have shown that VPS35 interacts with and sequesters Ku protein, a pivotal player in NHEJ pathway, through a dynamic interaction. Nuclear VPS35 disrupts the Ku-mediated activation of DNA-PKcs, thereby impeding the timely recruitment of essential repair factors like XLF and DNA-Ligase 4 to DNA damage sites, ultimately compromising the NHEJ efficiency. Moreover, our findings reveal a dynamic interplay between VPS35 and Ku protein in response to DNA damage. Upon the occurrence of DNA damage, the strength of the VPS35-Ku interaction weakens, prompting VPS35 to strategically relocate from the nucleus to the cytoplasm. This orchestrated relocation might effectively release Ku protein, allowing it to fulfill its indispensable role in orchestrating DNA repair processes.

Ku protein stands as a pivotal player in the NHEJ pathway, renowned for its paramount role as the initial DNA end-binding factor. Its exceptional ability to bind double-stranded DNA ends with high affinity, irrespective of sequence, stems from a central ring structure formed by the intricate intertwining of Ku70 and Ku80 subunits (Zahid et al. 2021). At the site of DNA breaks, Ku serves as a versatile scaffold, engaging in both direct and indirect interactions with several NHEJ factors and processing enzymes, thereby orchestrating the assembly of the entire DNA repair complex. Intriguingly, a diverse array of Ku-associated proteins have been identified, each contributing to modulating Ku’s interaction with DNA ends (Ghosh and Raghavan 2021). Among these, PAXX interacts with Ku via its C-terminus, which fosters the accumulation of Ku protein at DSBs, facilitating V(D)J recombination (Liu et al. 2017; Ochi et al. 2015; Seif-El-Dahan et al. 2023). Furthermore, ZNF384, often referred to as a “Ku-adaptor”, interacts with Ku70/Ku80 through its N-terminus, enhancing their assembly and subsequently promoting the recruitment of downstream NHEJ factors, such as APLF, thereby fine-tuning the repair process (Singh et al. 2021). Meanwhile, FOXL2, a forkhead family transcriptional regulator, exerts control over the choice of DSB repair pathways through acetylation-mediated binding to Ku (Jin et al. 2020). Upon DSB induction, SIRT1 translocates to the nucleus, deacetylating FOXL2 at lysine 124, which releases Ku70 and Ku80 from FOXL2, facilitating Ku complex formation (Jin et al. 2020). Intriguingly, FOXL2 ablation enhances Ku recruitment to DSB sites, perturbing the delicate balance of DSB repair kinetics by accelerating NHEJ while inhibiting HR, ultimately precipitating catastrophic genomic consequences (Jin et al. 2020). Our study demonstrates that VPS35 engages in an interaction with Ku protein, effectively sequestering it. Notably, upon the occurrence of DNA damage, VPS35 dissociates from Ku protein, thereby releasing it to perform its vital function in initiating the NHEJ pathway. These findings unveil an additional manner of regulatory control, enabling the fine-tuning of DNA repair efficiency.

FAM21, also known as WASH complex subunit 2 (WASHC2), mediates the targeting of the WASH complex to endosomes via a direct interaction between FAM21 C-terminal fragment and the retromer protein VPS35 within the cytoplasmic milieu (Jia et al. 2012). We have further illuminated a nuclear function of FAM21, wherein it localizes precisely to DNA damage sites through its interaction with the Ku protein (Wang et al. 2024). This orchestrated engagement enhances the recruitment of the WASH complex to these damaged sites, thereby bolstering the NHEJ efficiency (Wang et al. 2024). The interaction between VPS35 and FAM21 prompted us to delve into the potential contribution of VPS35 in the DNA repair machinery. Our findings, albeit confirming a functional role for VPS35 in DNA repair processes, reveal an intriguing independence from the FAM21/WASH complex in this regard. This functional decoupling is evidenced by three key findings: (1) laser micro-irradiation assays show VPS35 depletion does not hinder the recruitment of the FAM21/WASH complex to DNA damage sites (Fig. S5A-D); (2) biochemical analyses demonstrate VPS35 knockout does not perturb the established interaction between WASH and Ku (Fig. S5E); (3) genetic epistasis analyses reveals VPS35 knockout enhances NHEJ repair regardless of WASH status, while WASH knockdown impairs NHEJ repair irrespective of VPS35 expression, indicating non-overlapping NHEJ regulation of VPS35 and WASH. These observations suggest that VPS35 and WASH possibly regulate NHEJ through parallel pathways, with their opposing functional impacts arising from independent interaction with Ku protein.

In recent years, liquid-liquid phase separation (LLPS) has been identified to play crucial roles in numerous biological process that occurs locally, including DNA repair (Wang et al. 2023). During LLPS, biomolecules segregate into a highly concentrated dense phase and a concomitant dilute phase, in which dynamic material exchange takes place over short timescales (Gao et al. 2022; Sabari et al. 2018). Several DDR factors has been reported to undergo LLPS, including 53BP1 (Kilic et al. 2019), Poly(ADP-ribose) (Altmeyer et al. 2015) and Fused in sarcoma (FUS) (Hofweber et al. 2018), among others. By using micro-irradiation assay, we observed a limited recruitment of VPS35 to DNA damage sites (Fig. 3A and C). Notably, despite VPS35 interacts with Ku protein, depletion of Ku protein failed to affect the recruitment of VPS35 (Fig. 3E-F), indicating the recruitment of VPS35 is independent of Ku protein. Since VPS35 does not interact with DNA ends (Fig. 3G), it is plausible to infer that its recruitment might rely on a DNA interaction-independent mechanism, such as LLPS. Further investigation is still needed to identify the nuclear partners of VPS35 and decipher the mechanism underlying its recruitment to DNA damage sites.

VPS35 was reported to determine the fate of damaged mitochondrial genome (mtDNA) in the cytoplasm. The gradual accumulation of mutations in the mtDNA over time is a universal aging hallmark across tissues (Gonzalez-Freire et al. 2015). In addition to canonical forms of mitophagy, a VPS35 endosome required mitophagy process has attracted considerable attention as a crucial mechanism for eliminating dysfunctional mitochondrial DNA (Braschi et al. 2010; McLelland et al. 2014; Soubannier et al. 2012; Williams et al. 2018). Upon mtDNA damage, VPS35 associates with mitochondria, guiding nucleoid- and dsDNA-bearing particles towards the lysosomal compartment (Sen et al. 2022). This VPS35 recruitment is contingent upon SAMM50, which facilitates a targeted association specifically with nucleoid-rich regions upon mtDNA insult (Sen et al. 2022). In this study, our investigation has expanded upon previous reports, revealing that VPS35 not only governs the destiny of mitochondrial DNA (mtDNA) within the cytoplasm, but also intimately associates with the fate of damaged DNA residing in the nucleus. This underscores a pivotal role for VPS35 in the cellular management of damaged DNA across the entire cell.

While our findings emphasize the nuclear role of VPS35 in sequestering Ku to suppress DNA repair, the cytoplasmic PLA signals (Fig. 2D-E) suggest broader regulatory mechanisms that extend beyond DNA repair pathways. Ku proteins, though predominantly nuclear, exhibit dynamic subcellular shuttling, enabling their participation in cytoplasmic processes such as cytosolic DNA sensing and subsequent activation of innate immune responses (Sui et al. 2021; Tao et al. 2022). The interaction between VPS35 and Ku in the cytoplasm may fine-tune Ku’s availability for nuclear DNA repair or play a role in immune surveillance, potentially modulating the cellular response to viral infections or mitochondrial DNA leakage. This duality underscores the complexity of Ku regulation and highlights VPS35 as a potential coordinator of Ku’s multifaceted roles, warranting further exploration into its cytoplasmic contributions to DNA immunity.

Cancer cells frequently exhibit compromised DNA repair and DDR signaling, paradoxically enabling survival under genotoxic stress while accumulating mutations that drive heterogeneity (Luo et al. 2024; Wang et al. 2021). Utilizing the GEPIA2 database (http://gepia2.cancer-pku.cn), we observed that VPS35 is highly expressed in the majority of tumors relative to normal tissues (Fig.S10). Our findings reveal that VPS35 overexpression sequesters Ku proteins, delaying their recruitment to DNA lesions and impairing DNA repair capacity. In cancers with high VPS35 levels, this suppression of repair could create a permissive environment for mutation accumulation, accelerating tumor evolution. Notably, while defective repair typically sensitizes cells to genotoxic therapies, VPS35-overexpressing tumors might paradoxically enhanced survival signaling. This hypothesis aligns with studies showing that VPS35 promotes the proliferation of hepatocellular carcinoma, breast cancer and gastric cancer (Guo et al. 2021; Li et al. 2021; Zhang et al. 2020; Zhou et al. 2024). Specifically, VPS35 promotes the sorting and trafficking of transmembrane receptor, thereby augmenting the proliferation of hepatoma cell through the PI3K/AKT signaling pathway (Zhang et al. 2020). In line with this, another study demonstrated that the transcriptional activation of VPS35 by KLF7 propels hepatocellular carcinoma cells growth and metastasis by activating Ccdc85c-medicated β-catenin pathway (Guo et al. 2021). Similarly, VPS35 promotes the progression of breast cancer and is essential for autophagy completion (Li et al. 2021). Furthermore, in gastric cancer cells, VPS35 expression is upregulated, and it positively regulates the proliferation and peritoneal metastasis of gastric cancer cells through integrin/FAK/SRC signalling-mediated IL-6/STAT3 pathway activation in a YAP-dependent manner (Zhou et al. 2024). Collectively, VPS35 serves as a critical node linking genome stability and oncogenic signaling. Its overexpression in cancers creates a vicious cycle: suppressed DNA repair drives mutation accumulation, while enhanced retrograde trafficking sustains proliferative signaling, ultimately fueling tumor progression.

While our study identifies VPS35 as a regulator of Ku availability at DNA damage sites, the potential role of Ku70 post-translational modifications (PTMs) in this process remains unexplored. Emerging evidence demonstrates that phosphorylation of Ku70 mediates its dissociation from DNA ends to facilitate HR in S phase (Lee et al. 2015). Intriguingly, mice bearing non-phosphorylatable Ku70 mutants exhibit impaired HR efficiency, genomic instability and increased incidence of spontaneous hepatocellular carcinoma (Saha et al. 2021). Additionally, cyclin-Cdk-dependent phosphorylation of Ku70 during S-M phases regulates its interaction with replication origins (Mukherjee et al. 2016), while SUMOylation of Ku80 modulates DNA repair and chemoresistance in colorectal cancer (Feng et al. 2023). These findings collectively suggest that dynamic PTMs orchestrate Ku’s functional switching between chromatin interactions and repair pathways. Future studies employing PTM-deficient Ku70 mutants will clarify whether VPS35 sequestrates Ku from DNA damage sites through affecting the PTMs of Ku, offering further mechanistic depth to our findings.

While our data demonstrate that DNA damage triggers VPS35-Ku dissociation and VPS35’s nuclear-to-cytoplasmic translocation, the precise regulatory signals remain unclear. Future studies will explore whether PTMs (e.g., phosphorylation) or interactions with nuclear export machinery mediate this process, potentially linking VPS35’s dynamic localization to broader DNA damage response pathways.

In summary, this study demonstrates that VPS35 suppresses NHEJ DNA repair by interacting with the Ku protein and sequestering it away from DNA damage sites. Our findings reveal a novel regulatory layer in DNA repair capacity, wherein VPS35 modulates the NHEJ by spatially restricting Ku’s chromatin accessibility. Three critical mechanistic questions warrant further investigation: (1) the molecular basis of VPS35-mediated suppression of HR, (2) the DNA damage-responsive signaling which triggers VPS35-Ku complex dissociation, and (3) the spatiotemporal control of VPS35 recruitment to DNA lesions. Addressing these questions will provide new insights into the role and mechanisms of VPS35 in DNA repair.

## Supplementary Information

Supplementary Material 1: Figures S1–S10 and Table S1.

Supplementary Material 2: Table S2.

## Data Availability

Mass spectrometry analysis Data is provided within the supplementary information files Table S2.

## Abbreviations

DSBs: DNA double strand breaks
NHEJ: non-homologous end joining
HR: homologous recombination
DNA-PKcs: DNA-dependent protein kinase catalytic subunit
KAP1: KRAB associated protein 1
DDR: DNA damage response
XLF: XRCC4-like factor
PAXX: Paralog of XRCC4 and XLF
MRI: Modulator of retroviral infection
ERCC6L2: ERCC excision repair 6 like 2
FOXL2: Forkhead box protein L2
cGAS: cyclic GMP-AMP Synthase
WASH: Wiskott-Aldrich Syndrome Protein and SCAR Homolog
VPS35: Vacuolar protein sorting-associated protein 35
SHRC: WASH regulatory complex
NLS: nuclear localization signal
DTT: DL-Dithiothreitol
PBS: phosphate buffered saline
ANOVA: one-way Analysis of Variance
KO: knockout
gRNA: guide RNA
WT: wild-type
EtBr: ethidium bromide
PLA: proximity ligation assay
CDS: coding sequence
NLS.VPS35: NLS-tagged VPS35
WASHC2: WASH complex subunit 2
LLPS: liquid-liquid phase separation
mtDNA: mitochondrial DNA
RPA1: Replication protein A-1
