# Supplementary material for: Nuclear VPS35 attenuates NHEJ repair by sequestering Ku protein
Source: Mol Med. 2025 Jun 9;31:222. doi: 10.1186/s10020-025-01288-1 (PMC12147292; doi:10.1186/s10020-025-01288-1)
Supplement: Supplementary file 1 — Supplementary Material 1: Figures S1–S10 and Table S1. [file 10020_2025_1288_MOESM1_ESM.docx]

**Supplementary Materials**

**Nuclear VPS35 attenuates NHEJ repair by sequestering Ku protein**

Luping Zhang^1,2^, Yonghong Nie^1,2^, Tuo Tang^1,2^, Yanji Lu^1,2^, Wenlong Li^1,2^, Xian Hong^1^, Qiang Li^1,2^, Aixue Zheng^1,2^, Yongpei Li^1,2^, Jianwen Zhou^3^, Li Fan^3^, Tao Wang^1,2,^*, Zhihui Deng^1,4,^*

^1^Laboratory of Protein Structure and Function, Institute of Medicine and Pharmacy, Qiqihar Medical University, Qiqihar, Heilongjiang 161006, China.

^2^Department of Medical Technology, Qiqihar Medical University, Qiqihar, Heilongjiang 161006, China

^3^Laboratory of Molecular Biology, Institute of Medicine and Pharmacy, Qiqihar Medical University, Qiqihar, Heilongjiang 161006, China.

^4^Heilongjiang Provincial key Laboratory of Precise Diagnosis and Neuropsychological Regulation of Mental Disorders, Qiqihar, Heilongjiang 161006, China.

*Correspondence: wangtao@qmu.edu.cn (T.W.); deng.zhihui@qmu.edu.cn (Z.D.)


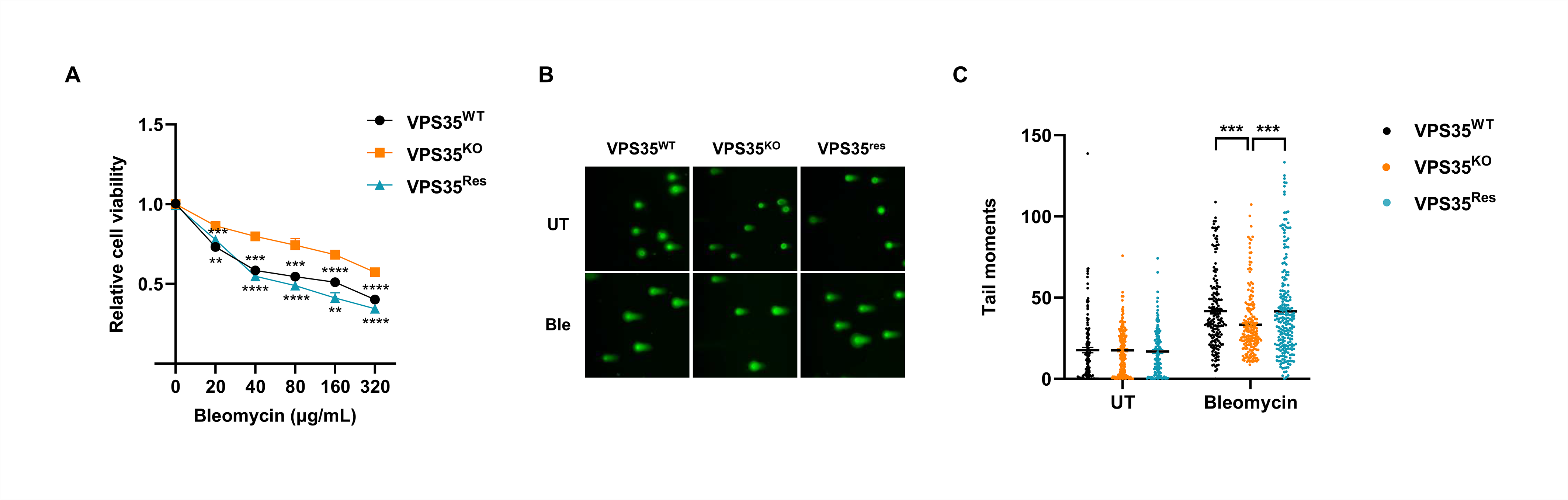


Fig.S1 Loss of VPS35 sensitize cells to bleomycin. (A) Cell viability of VPS35^WT^, VPS35^KO^ and VPS35^Res^ HeLa cells treated with indicated concentration of bleomycin for 48 h were measured with a CCK8 assay. Data are presented as mean ± SD. **, p < 0.01; ***, p < 0.001; ****, p < 0.0001. (B) VPS35^WT^, VPS35^KO^ and VPS35^Res^ HeLa cells treated with 200 μg/mL bleomycin for 60 min were allowed to recover for 30 min. Neutral Comet assay was performed to assess the DSB repair capacity. (C) Tail moments were quantified across three independent biological replicates, with 136-256 cells analyzed per experimental condition. Data are presented as mean ± SEM. ***, p < 0.001.


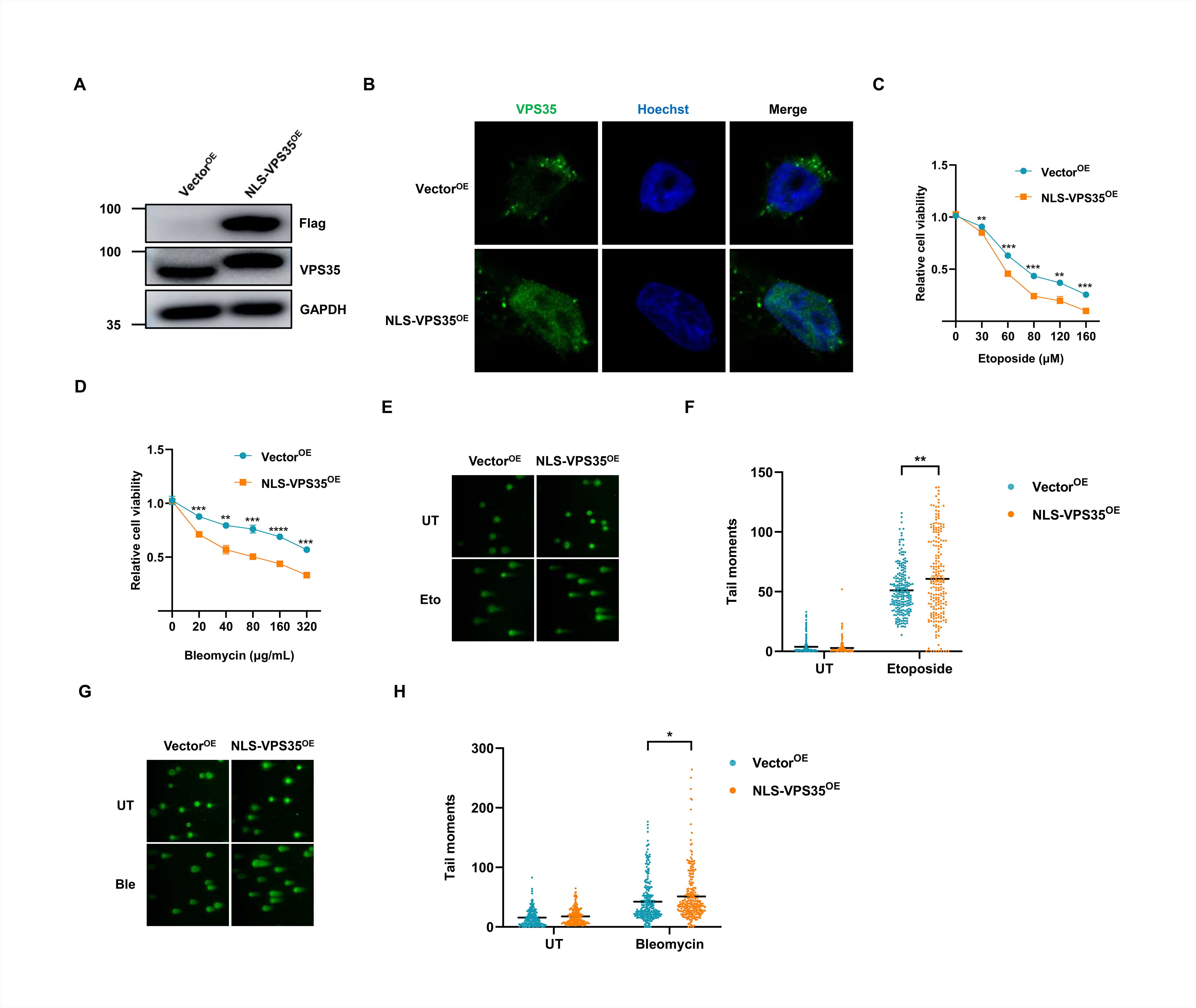


Fig.S2 NLS-VPS35 overexpression desensitized cells to DNA damage. (A) Generation of HEK-293T cells stably expressing vector (Vector^OE^) or NLS.VPS35 (NLS.VPS35^OE^). VPS35 expression in HEK-293T stable cell lines were measured by immunoblot. (B) Expression of VPS35 was examined by immunofluorescence in HEK-293T stable cell lines. (C) Cell viability of Vector^OE^ and NLS.VPS35^OE^ HEK-293T cells treated with indicated concentration of etoposide for 48 h were measured with a CCK8 assay. Data are presented as mean ± SD. **, p < 0.01; ***, p < 0.001. (D) Cell viability of Vector^OE^ and NLS.VPS35^OE^ HEK-293T cells treated with indicated concentration of bleomycin for 48 h were measured with a CCK8 assay. Data are presented as mean ± SD. **, p < 0.01; ***, p < 0.001; ****, p < 0.0001. (E) Vector^OE^ and NLS.VPS35^OE^ HEK-293T cells treated with 100 μM etoposide for 60 min were allowed to recover for 30 min. Neutral Comet assay was performed to assess the DSB repair capacity. (F) Tail moments were quantified across three independent biological replicates, with 230-256 cells analyzed per experimental condition. Data are presented as mean ± SEM. **, p < 0.01. (G) Vector^OE^ and NLS.VPS35^OE^ HEK-293T cells treated with 200 μg/mL bleomycin for 60 min were allowed to recover for 30 min. Neutral Comet assay was performed to assess the DSB repair capacity. (H) Tail moments were quantified across three independent biological replicates, with 195-256 cells analyzed per experimental condition. Data are presented as mean ± SEM. *, p < 0.05.


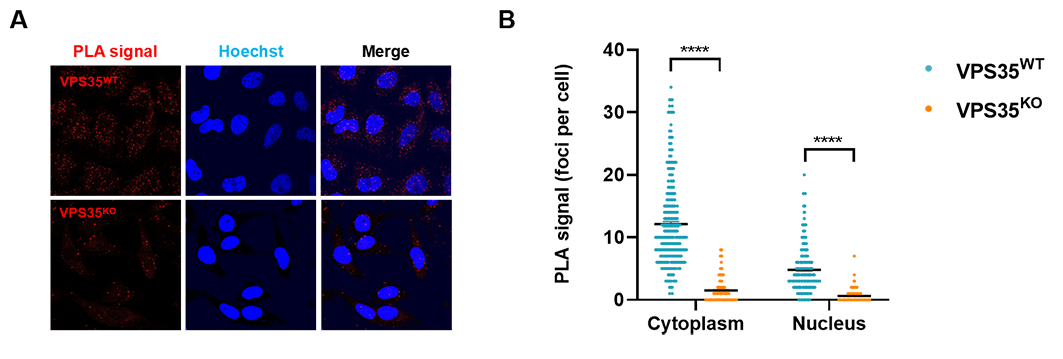


Fig.S3 VPS35 interacts with Ku80 in both the nucleus and the cytoplasm. (A) The interactions between VPS35 and Ku80 were assessed by PLA with the pairs of primary antibodies against VPS35 and Ku80 in VPS35^WT^ and VPS35^KO^ HeLa cells. Co-staining VPS35-Ku80 in VPS35^KO^ cells were used as negative control. (B) PLA signal foci numbers per nucleus in 256 cells of each group from three independent biological replicates were calculated and presented as mean ± SEM. ****, p < 0.0001.


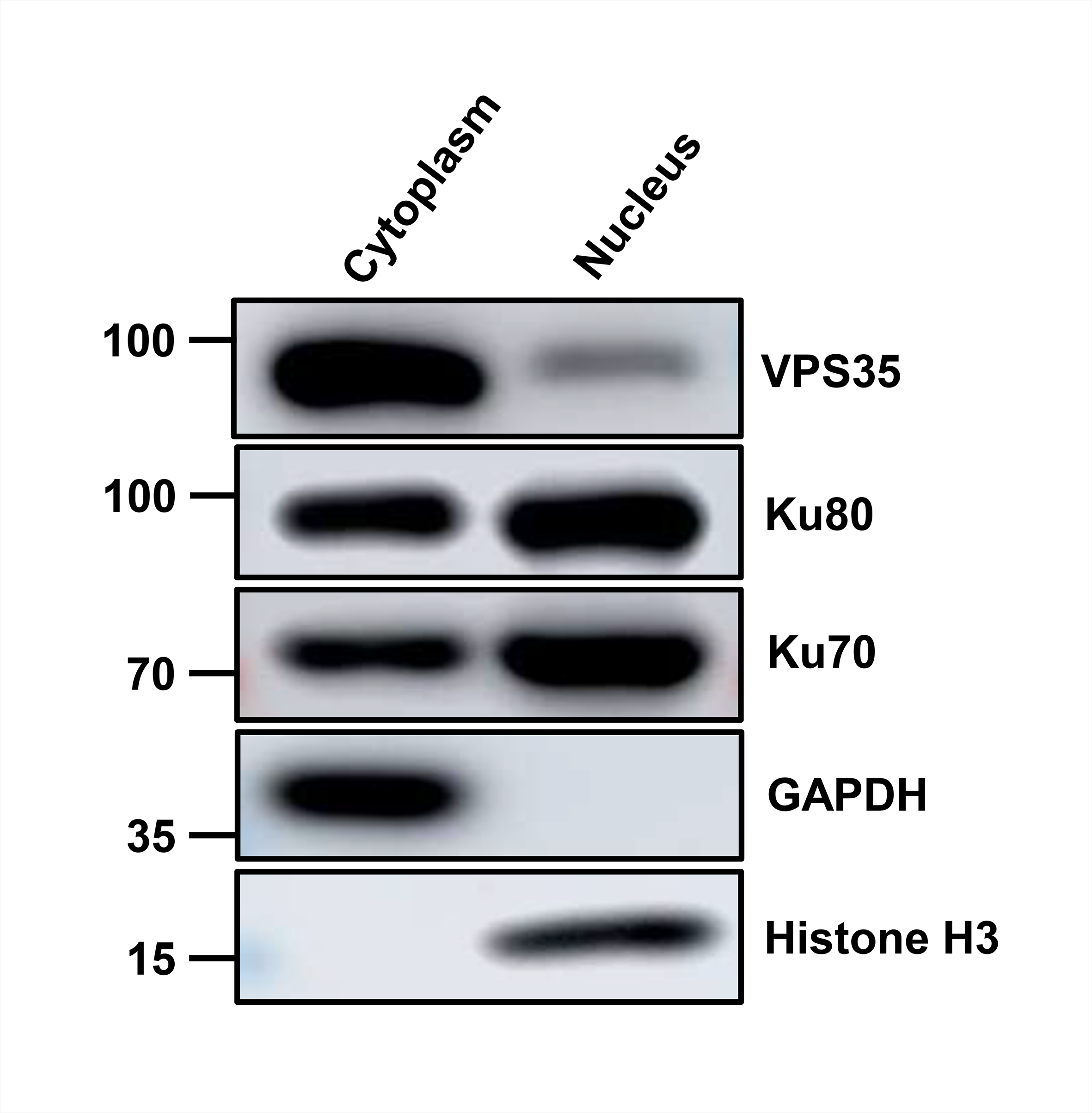


Fig.S4 The dual localization patterns of VPS35 and Ku protein. Fractionated cytoplasmic and nuclear extracts from HeLa cells were analyzed by immunoblotting with the indicated antibodies. GAPDH and Histone H3 serve as compartment-specific loading controls.


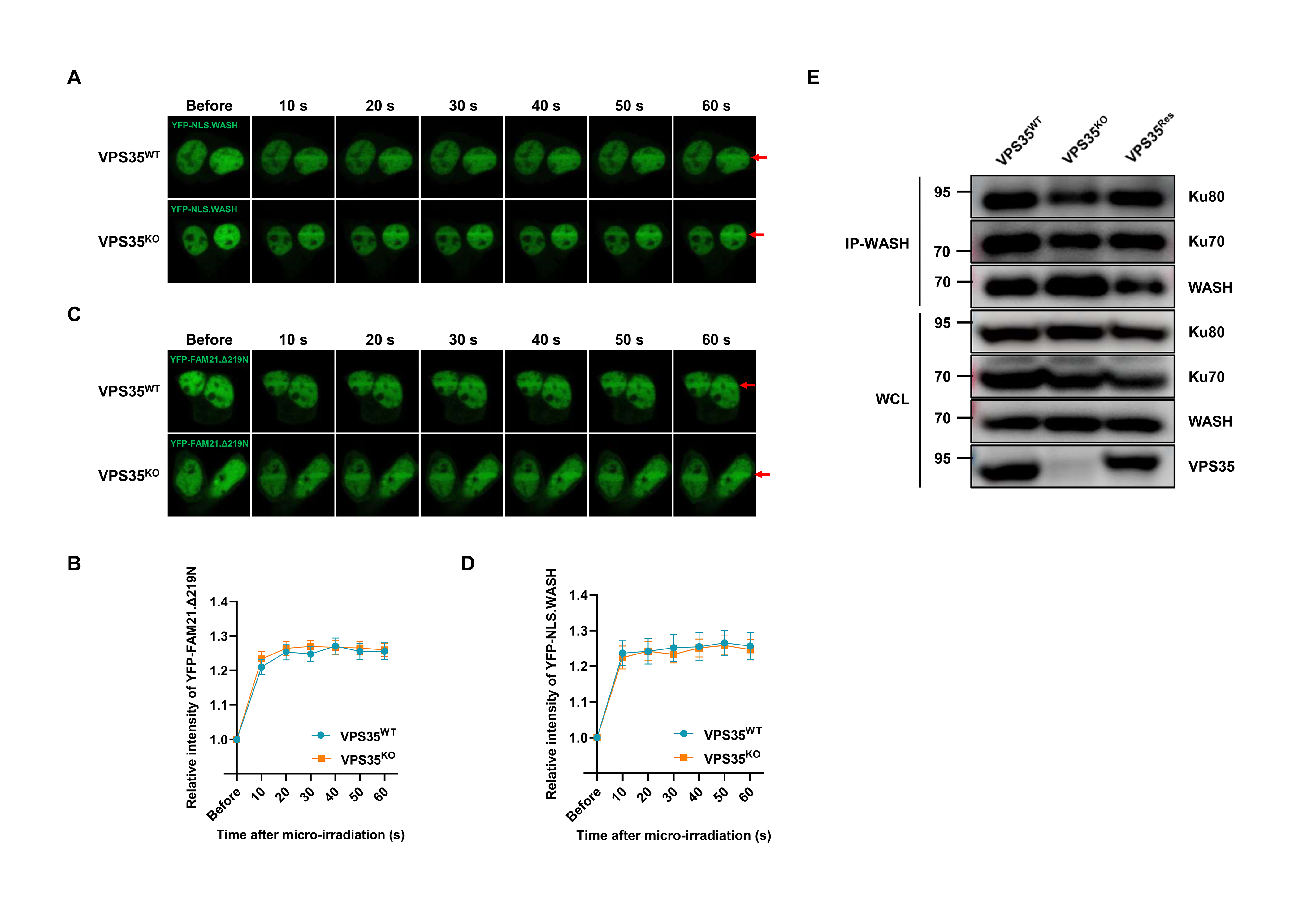


Fig.S5 VPS35 does not impact the recruitment of WASH/FAM21 to DNA damage sites. (A) Laser micro-irradiation was used to induce DNA damage in a line pattern in VPS35^WT^ and VPS35^KO^ HEK-293T cells transfected with YFP-NLS.WASH. (B) Quantification of WASH recruitment to DNA damage tracks in VPS35^WT^ and VPS35^KO^ HEK-293T cells. The relocation kinetics of nuclear WASH to DNA damage sites was monitored in a time course as indicated. (C) Laser micro-irradiation was used to induce DNA damage in a line pattern in VPS35^WT^ and VPS35^KO^ HEK-293T cells transfected with YFP-FAM21.Δ219N. (D) Quantification of FAM21.Δ219N recruitment to DNA damage tracks in VPS35^WT^ and VPS35^KO^ HEK-293T cells. The relocation kinetics of nuclear FAM21.Δ219N to DNA damage sites was monitored in a time course as indicated. (E) The cell lysates of VPS35^WT^, VPS35^KO^ and VPS35^Res^ HeLa cells were immunoprecipitated with anti-WASH antibody and analyzed by immunoblot.


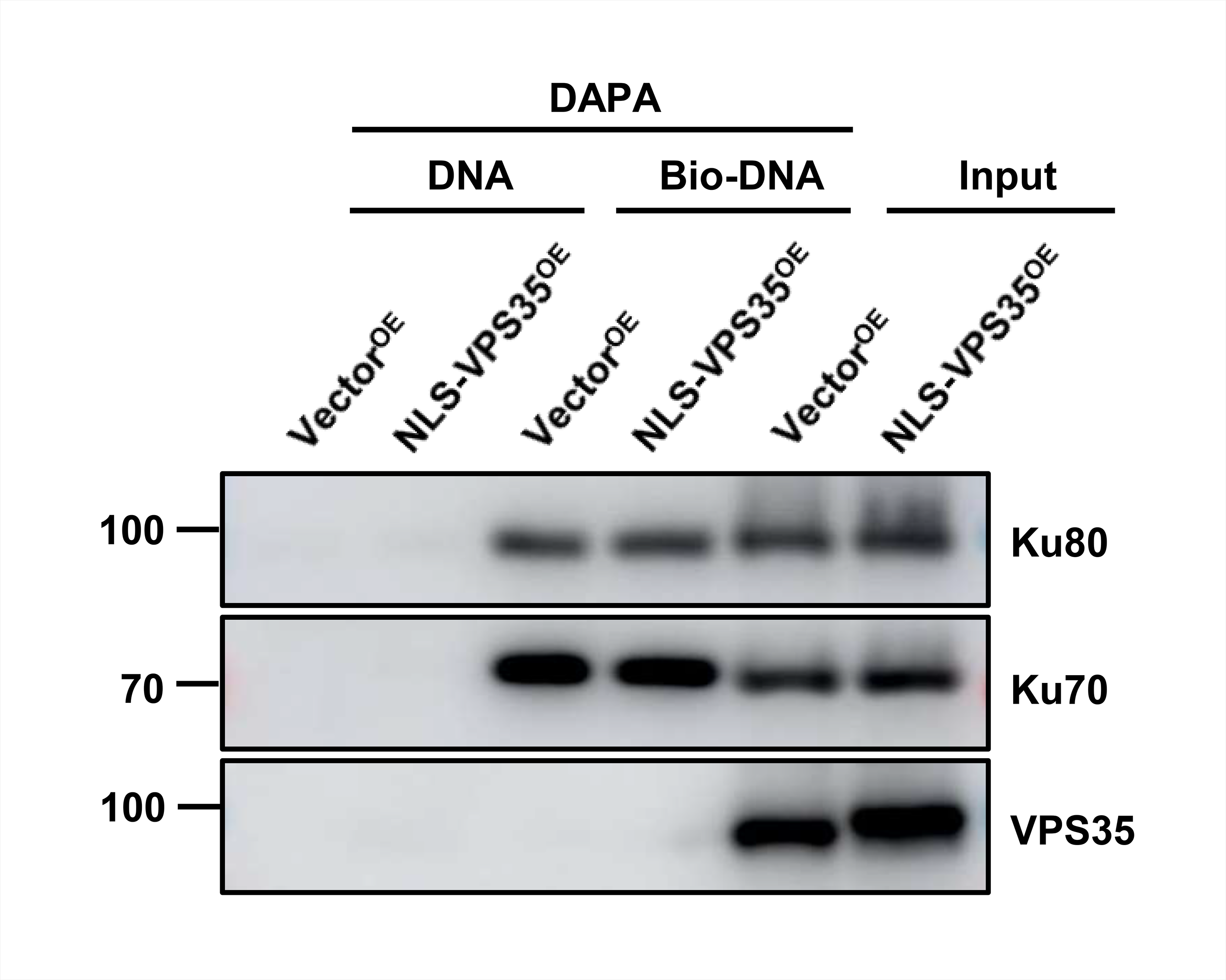


Fig.S6 VPS35 does not directly impair the DNA-binding capacity of Ku protein. Biotin labeled DNA were incubated with lysates from Vector^OE^ and NLS-VPS35^OE^ HEK-293T cells. DNA-bound proteins were captured using streptavidin beads and analyzed by immunoblotting for Ku70, Ku80, and VPS35.


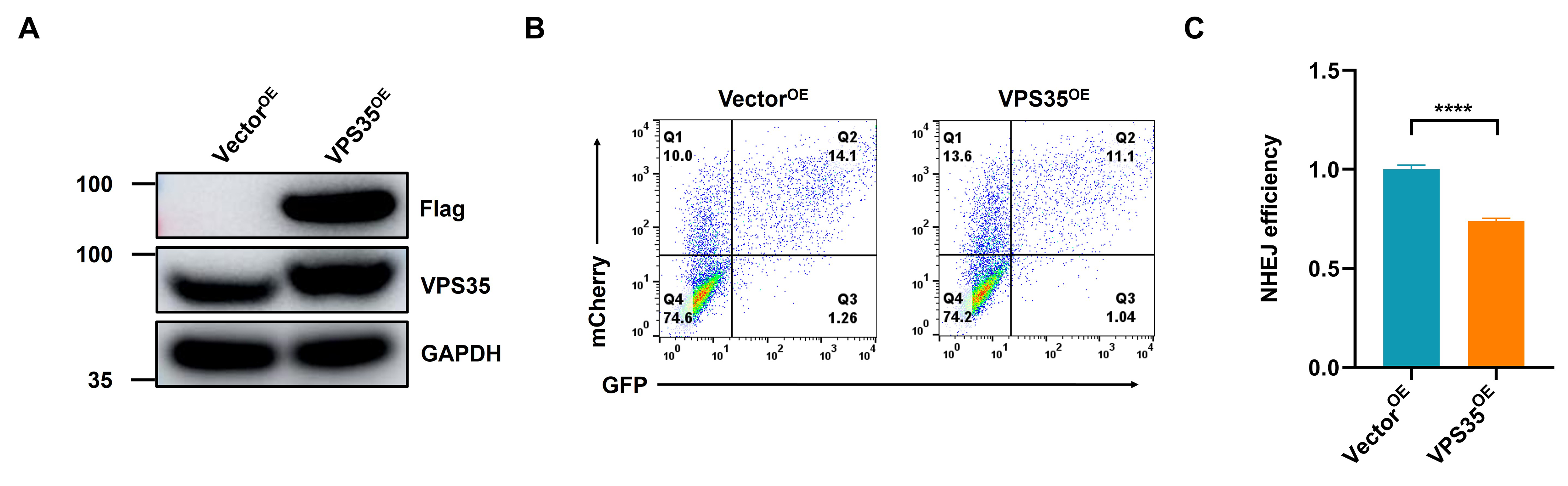


Fig.S7 Overexpression of VPS35 inhibits NHEJ efficiency. (A) Generation of HEK-293T cells stably expressing VPS35 (VPS35^OE^). (B) Vector^OE^ and VPS35^OE^ HEK-293T cells were subjected to NHEJ assay. (C) NHEJ efficiency was calculated by comparing the number of GFP-positive cells with the number of mCherry-positive cells. Data were presented as mean ± SD. ****, p < 0.0001.


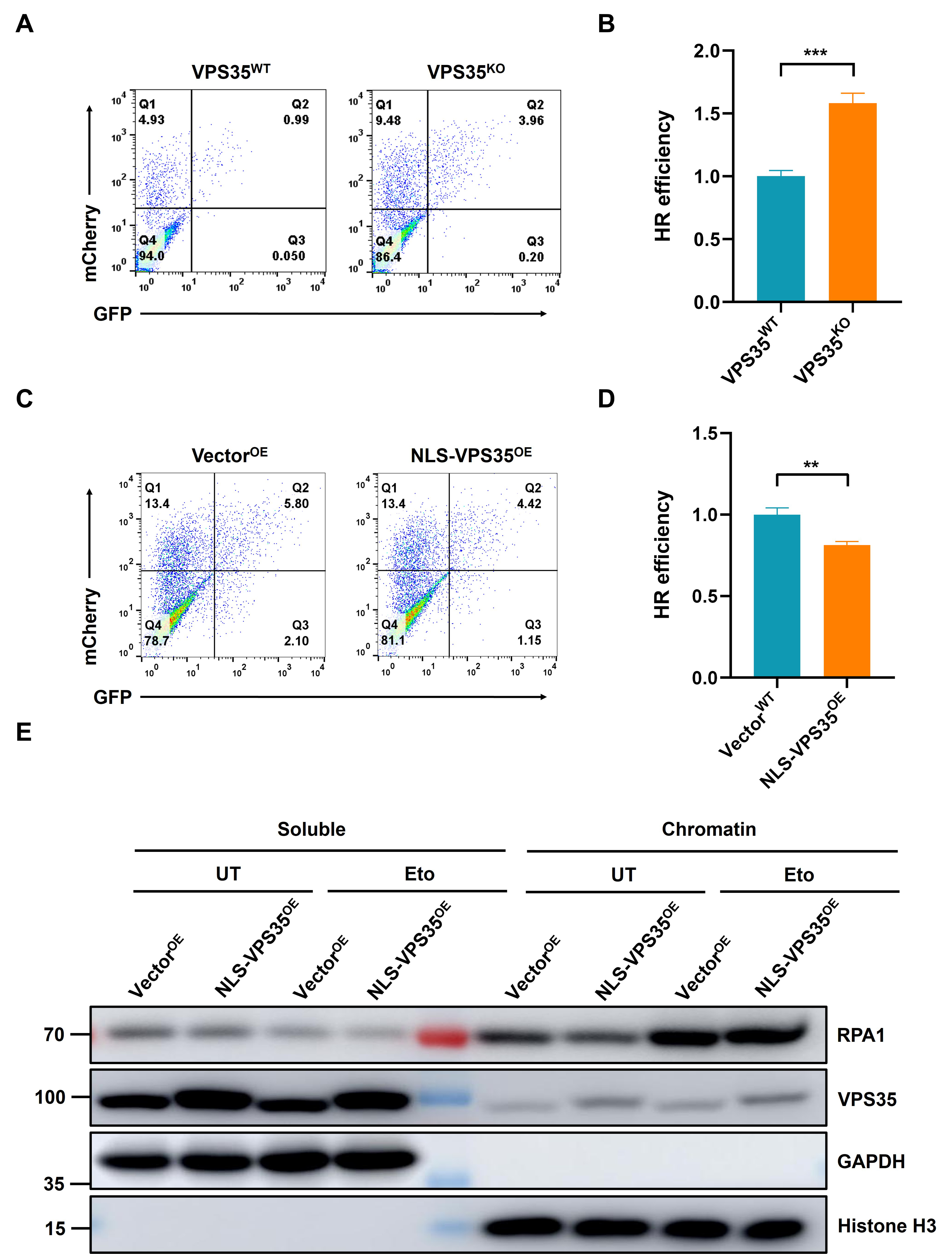


Fig.S8 VPS35 suppresses HR efficiency. (A) VPS35^WT^ and VPS35^KO^ HEK-293T cells were subjected to HR assay. (B) HR efficiency was calculated by comparing the number of GFP-positive cells with the number of mCherry-positive cells. Data were presented as mean ± SD. ***, p < 0.001. (C) NLS.VPS35^OE^ and Vector^OE^ HEK-293T cells were subjected to HR assay. (D) HR efficiency was calculated by comparing the number of GFP-positive cells with the number of mCherry-positive cells. Data were presented as mean ± SD. **, p < 0.01. (E) Vector^OE^ and NLS.VPS35^OE^ cells treated with or without 100 μM etoposide for 2 h were subjected to chromatin fractionation assay followed by SDS-PAGE and immunoblotting.


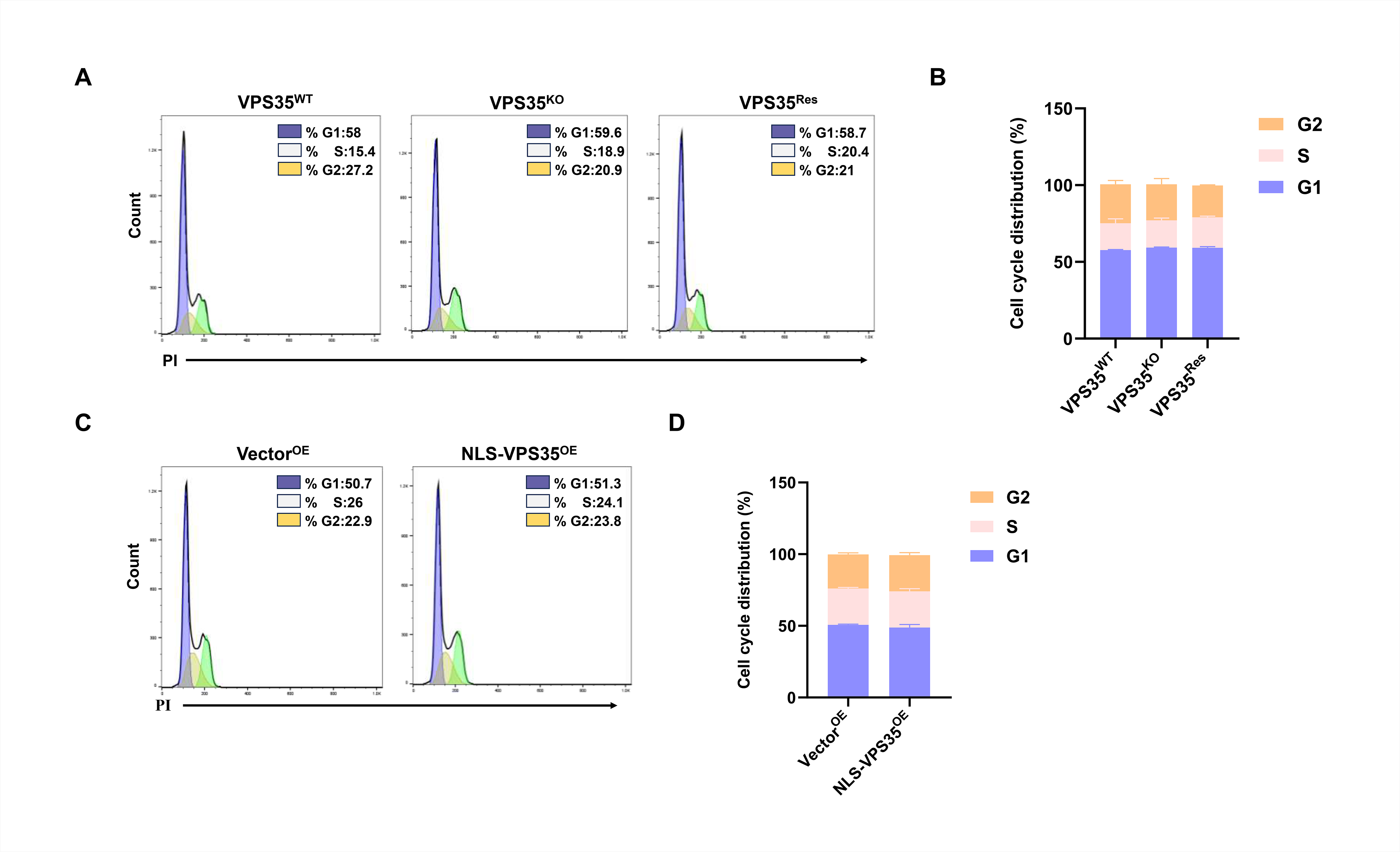


Fig.S9 VPS35 does not perturb cell cycle progression. (A) Flow cytometric DNA content profiles of VPS35^WT^, VPS35^KO^ and VPS35^Res^ HeLa cells stained with PI. (B) Quantified cell cycle phase distributions (G1/S/G2-M) showing comparable progression among VPS35^WT^, VPS35^KO^ and VPS35^Res^ HeLa cells. (C) Flow cytometric DNA content profiles of Vector^OE^ and NLS-VPS35^OE^ HEK-293T cells stained with PI. (D) Quantified cell cycle phase distributions (G1/S/G2) showing comparable progression between Vector^OE^ and NLS-VPS35^OE^ HEK-293T cells. All analyses utilized ≥10,000 events/sample processed through FlowJo software. Results are expressed as mean ± SD across three biological replicates.


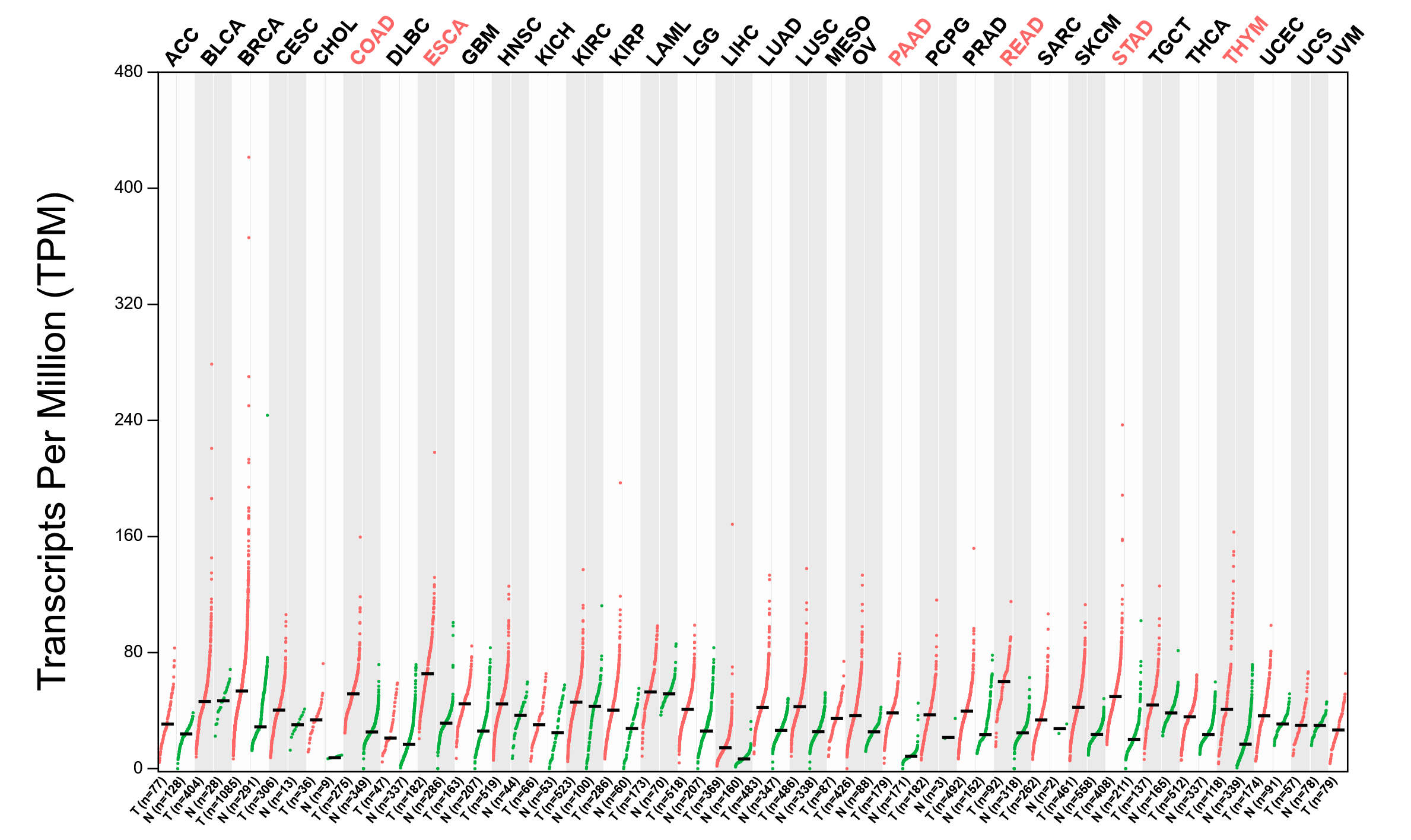


**Fig.S10 VPS35 is highly expressed in the majority of** **tumors relative to** **normal tissues.** The expression of VPS35 in various tumors and normal tissues were analyzed using GEPIA 2 dataset.

Table S1 Sequences of primers

| Primers | Sequence (5’ → 3’) | Application |
| --- | --- | --- |
| VPS35.Mlu.1.f | ATCGACGCGTATGCCTACAACACAGCAGTC | Construction of the plasmid PCI2.Flag.VPS35 |
| VPS35.Not1.r | ATAAGAATGCGGCCGCCTTAAAGGATGAGACCTTCAT | Construction of the plasmid PCI2.Flag.VPS35 |
| NLS.linker.VPS35. Xho1.f | GACTCTCGAGCCTAAGAAGAAGCGTAAGGTCGGAGGAGGAGGAAGTGGAGGAGGAGGAAGTGGAGGAGGAGGAAGTATGCCTACAACACAGCAGTCC | Construction of the plasmid pCMS3.H1p.shVPS35b/HA.YFP-NLS.linker.VPS35 |
| VPS35.Mlu1.r | GACTACGCGTTTAAAGGATGAGACCTTCATAAATTG | Construction of the plasmid pCMS3.H1p.shVPS35b/HA.YFP-NLS.linker.VPS35 |
| NLS.linker.VPS35.BamH1.f | CAGTGGATCCCCTAAGAAGAAGCGTAAGGTCG | Construction of the plasmid pLenti6.3.Flag.NLS.linker.VPS35 |
| VPS35.Xho1.r | CAGTCTCGAGTTAAAGGATGAGACCTTCATAAATTG | Construction of the plasmid pLenti6.3.Flag.NLS.linker.VPS35 |
| pG68.f | GCACCCCAGGCTTTACACTTTATG | Generation of a 572 bp duplex DNA |
| pG68.biotin.f | GCACCCCAGGCTTTACACTTTA/iBiodT/G | Generation of a biotinylated 572 bp duplex DNA |
| pG68.r | TGCGGCATCAGAGCAGATTG | Generation of a 572 bp duplex DNA or its biotinylated counterpart, pairing with primer pG68.f or pG68.biotin.f. |
